# Integrative taxonomy by molecular species delimitation: multi-locus data corroborate a new species of Balkan Drusinae micro-endemics

**DOI:** 10.1186/s12862-017-0972-5

**Published:** 2017-06-06

**Authors:** Simon Vitecek, Mladen Kučinić, Ana Previšić, Ivana Živić, Katarina Stojanović, Lujza Keresztes, Miklós Bálint, Felicitas Hoppeler, Johann Waringer, Wolfram Graf, Steffen U. Pauls

**Affiliations:** 1 Department of Limnology and Bio-Oceanography, Faculty of Life Sciences, University of Vienna, Vienna, Austria; 2 Department of Biology, Faculty of Science, University of Zagreb, Zagreb, Croatia; 3 Institute of Zoology, University of Belgrade-Faculty of Biology, Belgrade, Serbia; 4 Center for Systems Biology, Biodiversity and Bioresources, Faculty of Biology and Geology, Babeş-Bolyai University, Cluj-Napoca, Romania; 5 Senckenberg Biodiversity and Climate Research Centre BIK-F, Frankfurt’ to ‘Frankfurt am Main, Germany; 6 Institute of Hydrobiology and Aquatic Ecology Management, University of Natural Resources and Life Sciences, Vienna, Austria; 7 Senckenberg Research Institute and Natural History Museum, Frankfurt’ to ‘Frankfurt am Main, Germany

**Keywords:** Genetics, Phylogenetics, Trichoptera, Identification, New species, STACEY

## Abstract

**Background:**

Taxonomy offers precise species identification and delimitation and thus provides basic information for biological research, e.g. through assessment of species richness. The importance of molecular taxonomy, i.e., the identification and delimitation of taxa based on molecular markers, has increased in the past decade. Recently developed exploratory tools now allow estimating species-level diversity in multi-locus molecular datasets.

**Results:**

Here we use molecular species delimitation tools that either quantify differences in intra- and interspecific variability of loci, or divergence times within and between species, or perform coalescent species tree inference to estimate species-level entities in molecular genetic datasets. We benchmark results from these methods against 14 morphologically readily differentiable species of a well-defined subgroup of the diverse Drusinae subfamily (Trichoptera, Limnephilidae). Using a 3798 bp (6 loci) molecular data set we aim to corroborate a geographically isolated new species by integrating comparative morphological studies and molecular taxonomy.

**Conclusions:**

Our results indicate that only multi-locus species delimitation provides taxonomically relevant information. The data further corroborate the new species *Drusus zivici* sp. nov. We provide differential diagnostic characters and describe the male, female and larva of this new species and discuss diversity patterns of Drusinae in the Balkans. We further discuss potential and significance of molecular species delimitation. Finally we argue that enhancing collaborative integrative taxonomy will accelerate assessment of global diversity and completion of reference libraries for applied fields, e.g., conservation and biomonitoring.

**Electronic supplementary material:**

The online version of this article (doi:10.1186/s12862-017-0972-5) contains supplementary material, which is available to authorized users.

## Background

Species represent a fundamental information unit in biological research [1–3]. Species-specific abundance fluctuations integrated with autecological attributes are used to assess stream health and ecological water quality, evaluate the potential of disease and parasite vectors, and were found to be highly informative in species distribution modelling approaches [4–15]. Aggregate taxa, i.e., taxonomic entities comprising more than one species, often do not provide sufficient resolution to reap the power of ecological analysis [16–18]. Thus, estimation of, e.g., ecological water quality in compliance with the EU Water Framework Directive crucially depends on precise taxonomy to delineate and define species [16].

Given the taxonomic impediment – the worldwide decline of taxonomic competence to identify species based on morphological characters – biological sciences and policymaking are severely hampered by difficulties in compiling relevant and up-to-date baseline diversity data [19–21]. Indeed, taxonomy and assessment of eukaryotes remains primarily reliant on identification and comparison of morphological characters to define and address species [22–25]. However, characterization of species ideally uses different sources of information (ecological, morphological, anatomical, physiological, genomic, geographical or others) in an integrative taxonomic approach for the identification, delineation and description of taxa [2, 3, 26, 27]. Advances in molecular genetic methods recently promoted molecular taxonomy: species recognition and delineation based on unique genomic characters [28–30].

In parallel, a variety of methods for recognizing new species or testing species hypotheses was developed, usually referred to as ‘species delimitation tools’ [31–33]. However, as the methods are computationally demanding, analyses of multi-locus data sets are usually employed to disentangle few morphologically defined species [34–40]. Studies addressing many (>10) morphotaxa often use few methods and single locus data to corroborate identification and delineation of new taxa [41–44]. Thus, speciose taxa are less likely to be addressed in molecular species delimitation analyses.

Also, despite the demonstrated informativeness of molecular taxon delimitation to test species hypotheses, not all studies that successfully employ molecular taxonomic taxa delimitation tools follow through to describe these new taxonomic entities [3, 45, 46]. Interestingly, this has been related to the complex taxonomic procedures associated with the formal description of new species [45]. However it is more likely that molecular species hypotheses are ignored in integrative approaches due to insufficient morphological or ecological support (e.g., [47]).

In this contribution we benchmark results of molecular species delimitation against morphologically well-defined taxa in a highly diverse group of caddisflies. The subfamily Drusinae (Insecta, Trichoptera) constitutes an ideal model taxon to assess potential congruence of traditional and molecular taxonomic methods. This group of mostly cold-stenotopic species inhabiting Eurasian mountain ranges exhibits highly disjunct distribution patterns and high levels of micro-endemism, indicative of small-scale allopatric diversification and persistence of isolated lineages over geological time [48–52]. However, historic introgression – a process that complicates species delimitation – was demonstrated in some species of Drusinae [49]. In the Western Balkans, taxonomic richness of Drusinae is particularly high [50, 51, 53–58] and was presumably shaped by multiple glacial cycles and karstification (cf. [48, 51, 59]). Additionally, Western Balkan Drusinae are morphologically distinct with multi-locus molecular data showing minimal differences which potentially result from recent speciation [51, 59]. Together, these conditions make Drusinae a good model for testing the suitability and precision of species delimitation methods.

Here, we assess taxonomic informativeness of four recently developed exploratory molecular species delimitation tools by inferring entity richness hypotheses on a 3798 bp, 6 loci (mtCOI5-P, mtCOI3-P, 16S mrDNA, CADH, WG, 28S nrDNA), 14 morphospecies dataset comprising 1 new and 1 recently described species. As a test case, we aim to clarify the systematic status of a morphologically distinct potential new species and corroborate its distinctiveness in an integrative taxonomic approach. We predict that only methods directly integrating information from several loci will provide taxonomically conclusive results.

## Methods

### Collection and taxonomic methods

Adult specimens were collected using sweep nets, larvae were collected by hand-picking. Collected specimens were stored in 96% EtOH. Specimens were cleared for genitalic dissections and examinations using either the Qiagen Blood and Tissue Kit for DNA-extraction according to the manufacturer’s recommendation and subsequent KOH-treatment [60], or KOH-treatment. Nomenclature of male terminalia follows [61] (for *Limnephilus flavicornis* Fabricius) using the simplifying terms “superior appendages” for the lateral processes of segment X (cerci sensu [62]), and “intermediate appendages” for the sclerite and the anterior process of segment X (paraproct sensu [62]). Illustrations were prepared according to [63] in which pencil drawings made with a camera lucida are digitized, edited and inked in Adobe Illustrator (v. 16.0.4, Adobe Systems Inc.).

Morphology-based delimitation of species-level taxa was achieved in a classical comparative taxonomic approach: we scrutinized as many specimens as possible from as many populations as possible from as many Drusinae species as possible to discriminate intraspecific from interspecific variation in more than 300 adult and larval features (e.g., structure of male and female terminalia, wing venation, larval feeding apparatus, and larval pronotum shape; [64]) and unequivocally assigned specimens to species-level taxa (cf. [50, 55–58, 64]).

### Molecular methods

Whole genomic DNA was extracted from the abdomen or the thorax of adult or larval specimens using the DNEasy Blood and Tissue Kit (Qiagen) according to the manufacturer’s protocol. Standard PCR procedures and primers were used to amplify three mitochondrial gene regions (mtCOI5-P, mtCOI3-P, 16S rDNA [“mt16S”]) and three nuclear gene regions (nuCADH, nuWG [“nuWnt1”], 28S nrDNA [“nu28S”]) as previously described [64]. This combination of loci was chosen based on their demonstrated informativeness for phylogenetic inference, phylogeography, and prior successful usage in integrative taxonomic approaches in Trichoptera (e.g., [48–51, 57, 64–68]). PCR reactions were set up in 10 μl reactions. Unpurified PCR products were sequenced on an ABI 3177XL capillary sequencer at BiK-F using the PCR primers and two additional internal primers for 28SrDNA (D2UP-4 and D2DN-B; [67]). Sequences were edited in Geneious R6 (https://www.geneious.com/; [69]) and aligned using MAFFT v7 [70] as implemented in Geneious R6. The final dataset comprised 40 Drusinae specimens assigned to 14 morphological species, and three outgroup specimens (*Anisogamus waringeri*: fAns0101L; *Melamphophylax austriacus*: fMelaus0101M, fMelaus0102F) (Additional file 1).

### Phylogenetic inference and molecular species delimitation

Nucleotide substitution models for each partition were selected according to the Bayesian Information Criterion in the model test module of Mega v6 [71] (Table 1). For phylogenetic analysis, the mt16S and nu28S fragments were not partitioned; protein coding genes (mtCOI5-P, mtCOI3-P, nuCADH, nuWnt1) were partitioned by codon position.

**Table 1:** Substitution models used in phylogenetic analysis

Fragment	Unpartitioned	Codon position 1	Codon position 2	Codon position 3
mtCOI5-P	−	TN93 + I	HKY	GTR + G
mtCOI3-P	−	TN93 + I	JC	TN93 + G
mt16S	JC	−	−	−
nuCADH	−	HKY	HKY	HKY + I
nuWG	−	JC	JC	HKY
nu28S	T92 + G	−	−	−

Single gene phylogenies were estimated by Bayesian Inference through BEAST 2 [72] (5 × 10^9^ generations, sampling every 10,000th generation). Analyses were run 4× independently to assure topological convergence. BEAST log files were examined in Tracer v1.6 [73] to assess when runs had reached a stationary phase. A maximum clade credibility tree was estimated via TreeAnnotator v1.8.1 [74] based on the sampled trees after discarding the first 30% as burn-in. Also, congruence of phylogenetic signal among data partitions was assessed by examining ≥0.95 posterior probability topologies of single gene analyses.

A species trees was estimated using *BEAST [75] as implemented in BEAST 2 using unique specimen identifiers as the species trait, i.e. without a priori species definitions. We ran species tree analysis assuming a Yule speciation tree prior for 5 × 10^8^ generations 4× independently, sampling every 10,000th generation. *BEAST log files were examined in Tracer v1.6 to assess if runs had reached a stationary phase and converged on model parameters; maximum clade credibility trees were estimated as described above.

We then performed molecular species delimitation using tools complying with the following set of criteria: (1) the method was designed as naive exploratory tool without a priori assignment of specimens to groups or assumptions about relationships between specimens (we consequently excluded BPP [76], as this method requires at least an a priori clustering of specimens into groups and a guide tree); (2) the method was originally designed for molecular species delimitation (we consequently excluded applications like Brownie [32, 77], or SpeDeSTEM [78–80]); (3) the method was designed to exploit ‘standard’ partial genetic sequence data.

To assess potential congruence of molecular species delimitation methods, we performed a series of analyses employing several analytical resources selected as described above and report results against the benchmark of 14 morphologically identifiable species:

(I) Automatic Barcode Gap Detection [ABGD] was performed for each locus and a concatenated sequence data set via the ABGD webmask [81]. ABGD is a tool designed to infer species hypotheses based on automatized identification of barcode gaps between inter- and intraspecific pairwise distances in partial sequence data sets. The method does not make assumptions about data structure or evolutionary history, and only requires input data (a single locus alignment) to be sufficiently variable. Pairwise distances are computed either as simple p-distances, or as substitution-corrected distances (via either JC69 [82], or K2P [83] models). Barcode gaps are discovered as slope maxima of a function describing the relation of pairwise distance ranks and pairwise distances. The method partitions specimens into groups in a recursive manner in which every group is split again, until no further splits are possible, while integrating user-provided priors on maximum and minimum intraspecific differentiation and barcode gap width. The prior on intraspecific divergence (denoted *P* in the original publication and the software [81]) defines the threshold between intra- and interspecific pairwise distances, and is iterated from minimum to maximum through a user-defined number of steps [by default 10 steps from *P*_*min*_ = 0.001while *P*_*max*_ = 0.1]; the prior on barcode gap width (denoted *X* in the original publication and the software [81]) defines sensitivity of the algorithm as scaling factor of empiric maximum intraspecific divergence to estimate minimum barcode gap width between intra- and interspecific distances [by default *X* = 1.5]. From the thus provided set of possible partitions (up to 10 using default settings) Puillandre et al. [81] suggest to select the most plausible one(s) and assess their potential informativeness in an integrative approach. To avoid oversplitting of single species, we used the default distance metric (JC69) as this was previously found to produce more conservative species hypotheses [84, 85]. Likewise, we used the default minimum barcode gap width prior to derive relatively conservative species hypotheses — modifications (i.e., increasing or decreasing the default value) thereof were previously reported to either increase or decrease numbers of species hypotheses proposed, with smaller *X* values generally leading to more delimited entities and vice versa [84, 85]. Assuming that the potential barcode gap space would be satisfyingly described, we used default intraspecific divergence minima and maxima, and report delimitations at partition maximum as identified through recursive partitioning. Following the suggestions by Puillandre et al. [81], we only present species hypotheses at partition maxima as these correspond most closely to expected numbers of taxa.

(II) We used the GMYC [Generalized Mixed Yule Coalescent] model [86, 87], implementing single and multiple thresholds via the ‘splits’ package in R 3.2.1 [88, 89] on single gene trees and a *BEAST species tree to infer GMYC species. The GMYC model aims to discern stochastic birth-death processes (effectively a pure-birth Yule model) between species from neutral coalescent processes within species by analysis of time intervals between branching events (which, in turn, can be summarized as combination of independent Poisson processes) in time-calibrated single gene trees. Input prerequisites require a well-sampled, well-estimated, ultrametric single neutral locus tree that ideally represents the true species genealogy in absence of population structure and population size fluctuation. The method defines sets of species hypotheses based on single or multiple threshold times that potentially distinguish coalescent events from speciation events, and searches for a single maximum likelihood model of mixed speciation and diversification processes across the search space, i.e., sets of species hypotheses. Species hypotheses thus delineated correspond to the phylogenetic species concept.

(III) We analyzed single gene trees and a *BEAST species tree using the PTP [Poisson Tree Processes] model via the PTP webmask [76] using both heuristic ML and Bayesian implementations of the PTP algorithm. Somewhat similar to the GMYC model, the PTP model aims to discern speciation processes among species from diversification processes within species, but analyses numbers of substitutions between branching events instead of time intervals. Input prerequisites enforce the same assumptions as the GMYC model, but this method does not require an ultrametric input tree to delineate entities corresponding to the phylogenetic species concept. This delineation is instead achieved by heuristically inferring species delimitations and searching for a delimitation pattern that maximizes likelihood of a mixed model describing speciation and diversification processes as two independent Poisson process classes across the search space, i.e., sets of species hypotheses. Removing the outgroup in initial runs did not affect delimitation results; we consequently did not use this option.

(IV) We performed combined species tree estimation and species delimitation analysis as available via *Species Tree And Classification Estimation, Yarely* [STACEY] in BEAST 2 [90]. Simplified, this method (as its predecessor, DISSECT [91]) is an extension of the multispecies coalescence model used in *BEAST [75], in which a birth-death-collapse model is used to estimate the species tree [90, 91]. Further, specialized operators are included that model population sizes along branches, prune and regraft subtrees, modify node heights, and merge tips to minimal clusters. The method aims to maximize tree likelihood over a Bayesian tree space by using a MCMC model in which single tips can be merged to minimal clusters to estimate a *species or minimal cluster* (SMC) tree, while specific priors ensure compatibility between species and gene trees [90]. User-supplied priors define behaviour of MCMC moves, and provide a probability space for the expected number of species primarily through the Collapse Weight prior in combination with a Collapse Height parameter [90, 91]. While not extensively tested, a wide range of values [1e-4–1e-7] for Collapse Height was found to provide similar species delimitation results [91]. Contrastingly, the Collapse Weight prior was found to confound delimitation results if fixed, which can be circumvented if estimated during the MCMC process [90, 91]. Here, we assess importance of prior space settings for growth rate and population size scaling parameters in STACEY analysis, and test influence of ploidy settings on species delimitation results.

Initially, we assumed a birth-death speciation tree prior while using a Collapse Height of 0.0003, and estimated Collapse Weight with an initial value of 0.5 using a beta prior (1,1) around [0,1]; following suggestions for prior choice in species tree analysis using *BEAST [92], Jeffreys prior was used for growth rate and population scaling factor; the relative death was estimated using a beta prior (1,1) around [0,1]. We used equal ploidy settings, following results and arguments presented in [93–95]. We chose this approach to avoid disproportionate influence from mitochondrial partial sequence data and, consequently, treat each gene tree as likely as any other to diverge from the species tree. We did not modify the Collapse Height as preliminary experimental runs confirmed the patterns described in Jones et al. [91]: we found that values larger than or equal to 1e-3 lead to merging of all included specimens to very few (1–2) groups. Further, the NodeReheight operator was set to 3× its value as suggested by [90]. Genealogical relationships were estimated via STACEY 4× independently (1 × 10^7^ generations, sampling every 5,000th generation) after incorporating suggestions obtained from an initial run. STACEY log files were examined as stated above. Support for tree topologies estimated by STACEY were examined by constructing a maximum clade credibility tree running TreeAnnotator v1.8 after discarding the first third of all estimated trees. Species delimitations based on trees estimated by STACEY were assessed using speciesDA ([96]), using the same burn-in, a collapse height of a tenth of the average branch length (corresponding to a value of 0.0005), and default similarity cut-off. We conducted additional analyses to explore the sensitivity of the method to ploidy settings, and prior space for growth rate and population scaling factor. We used different combinations of ploidy settings for mitochondrial loci (using either the same value as for nuclear loci, or ¼ of the value used for nuclear loci which is commonly used in species tree analysis), Jeffreys priors, and logarithmic normal priors for growth rate and population scaling factor. Logarithmic normal priors were used to estimate both parameters, where one set mimicked empirical posterior distributions of growth rate and population scaling factor (where growth rate M = 2.5, S = 1.2, and population size M = −8.5, S = 2, respectively) while the others covered a prior space around M∈ [3, 5, 7], S∈[1.5, 2.5, 3.5] (resulting in nine possible prior combinations). Further, we edited ploidy settings in the original setup file and re-ran the original STACEY analysis to check for congruence with the new version as we noticed version-dependent differences between setup file templates. These analyses were run for 10 × 10^9^ generations, sampling every 10,000th generation, and analysed as described above. In total we thus tested the same data set with 24 prior setting combinations (cf. Table 3.)

Both GMYC and PTP methods were run on single gene trees estimated via BEAST exclusively, as taxa delimitations using both methods on BEAST trees were found to be consistent [97]. Further, both methods were used to estimate taxa delimitations on a *BEAST species tree. The practice of inferring species hypotheses on a species tree estimated through *BEAST represents a violation of many of the assumptions that lay the base for both the GMYC and the PTP model and we strictly advise against this approach. Here, however, we took this measure to allow for a more comprehensive comparison of species delimitation methods.

## Results

### Properties of the molecular dataset

Final alignments of mtCOI5-P (658 bp), mtCOI3-P (541 bp), mt16S (365 bp), nuCADH (848 bp), nuWnt1 (346 bp), and nu28S (1040 bp) comprised 27.65%, 29.76%, 15.34%, 12.97%, 23.98%, and 1.73% variable sites, respectively. Further, these alignments comprised 23.38%, 25.32%, 12.60%, 10.85%, 14.16%, and 1.25% parsimony-informative sites, respectively.

### Performance of molecular species delimitation methods

**Fig. 1:**
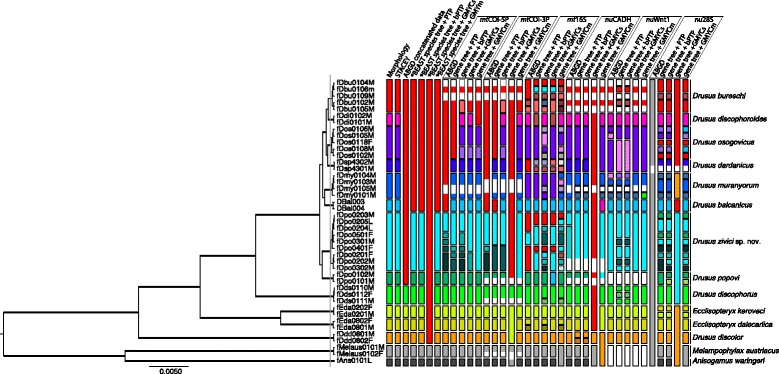
Summary of results of molecular species delimitation via ABGD, GMYC, PTP and STACEY methods. Analyses referring to STACEY and *BEAST are based on multi-locus datasets; for further analytical details, see text. Results are displayed relative to a STACEY species or minimal cluster tree (collapse height of 0.0005 as used in species delimitation analysis through speciesDA indicated by *vertical grey line*), and morphologically distinguishable taxa. Colouration indicates group membership of specimens; absence of colouration indicates missing data

Results from species-delimitation methods were found to be incongruent (Fig. 1, Table 2). Benchmarked against morphologically differentiable taxa, the majority of methods did not recover relevant species hypotheses. Only STACEY produced estimates corresponding to morphologically differentiable entities.

**Table 2:**
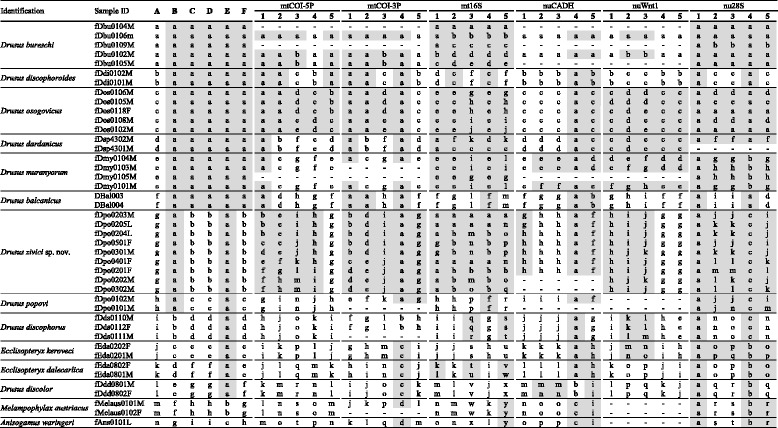
Tabular summary of results of molecular species delimitation via ABGD, GMYC, PTP and STACEY methods

Specimens referred to by unique identifiers are grouped by species; results are displayed relative to morphologically distinguishable taxa where identical minuscules indicate group membership as inferred through molecular species delimitation methods, where each column represents an independent analysis. Grey cells indicate conflicts of species hypotheses and the taxonomic benchmark, white cells indicate congruence of morphological delimitation and molecular species delimitation. Results of single-locus species delimitation methods are grouped by locus. Labels: A, results of STACEY analysis; B, results of ABGD analysis of a concatenated sequence dataset; C, results of PTP analysis of a *BEAST species tree; D, results of bPTP analysis of a *BEAST species tree; E, results of single threshold GMYC analysis of a *BEAST species tree; F, results of multiple threshold GMYC analysis of a *BEAST species tree; 1, results from ABGD analysis; 2, results from PTP analysis; 3, results from bPTP analysis; 4, results from single threshold GMYC analysis; 5, results from multiple threshold GMYC analysis.

For analytical details, see text. Order of specimens corresponds to order of specimens in Fig. 1. N-dash indicates missing data

#### Automated barcode gap analysis

ABGD based on single loci does not consistently propose species hypotheses. Based on the barcode region (mtCOI-5P), ABGD suggests 13 groups [7 morphological species] (aggregating *Drusus bureschi* + *D. discophoroides* + *D. osogovicus* + *D. dardanicus* + *D. muranyorum* + *D. balcanicus*; overpartitioning *Drusus zivici* sp. nov.), and suggests *D. popovi* and several *D. zivici* sp. nov. clades as different entities. Barcode gap analysis of partial mtCOI-3P sequence data suggests 11 groups [7 morphological species] (aggregating *D. bureschi* + *D. discophoroides* + *D. osogovicus* + *D. muranyorum* + *D. balcanicus*) overpartitioning *D. zivici* sp. nov.), and suggests *D. popovi* and and two *D. zivici* sp. nov. clades as different entities. ABGD analysis of partial mt16S sequences returns 15 groups [7 morphological species] (aggregating *D. bureschi* [partly] + *D. dardanicus* + *D. zivici* sp. nov. [partly] and *D. osogovicus* + *D. muranyorum*; overpartitioning *D. bureschi* and *Ecclisopteryx dalecarlica*), and suggests *D. popovi* differentiated from several *D. zivici* sp. nov. entities. Automated barcode gap analysis based on partial nuCADH sequence data suggests 15 groups [13 morphological species] (overpartitioning *Drusus zivici* sp. nov.), and suggests *D. popovi* and two *D. zivici* sp. nov. clades as different entities. Barcode gap analysis of the partial nuWnt1 sequence dataset in which data are missing for *D. popovi*, *Melampophylax austriacus*, *Anisogamus waringeri* suggests 12 groups [7 morphological species] (overpartitioning *D. osogovicus*, *D*. *muranyorum*), but is not informative regarding the status of *D. popovi* and *D. zivici* sp. nov. ABGD based on partial nu28S sequence data using default settings does not delimit any entities and is therefore not meaningful.

#### Generalized mixed Yule coalescent approach

GMYC results based on a single gene tree of partial mtCOI-5P sequence data suggests 16 groups [12 morphological species] (overpartitioning *D. osogovicus* and *D. zivici* sp. nov.) using the single threshold method, suggesting *D. popovi* and two clades of *D. zivici* sp. nov. as different entities — and 14 groups [11 morphological species] (aggregating (*D. bureschi* + *D. discophoroides*) overpartitioning *D. osogovicus*) using the multiple threshold method, suggesting *D. popovi* and *D. zivici* sp. nov. as different entities. Results of GMYC analysis based on a single gene phylogeny of partial mtCOI-3P sequence data suggests 4 groups [1 morphological species] (aggregating (*D. bureschi* + *D. discophoroides* + *D. osogovicus* + *D. dardanicus* + *D. muranyorum* + *D. balcanicus* + *D. zivici* sp. nov. + *D. popovi*), (*E. keroveci* + *E. dalecarlica* + *D. discolor*), and (*M. austriacus* + *A. waringeri*)) using the single threshold method, rejecting *D. popovi* and of *D. zivici* sp. nov. as different entities — and 13 groups [12 morphological species] (aggregating (*D. zivici* sp. nov. + *D. popovi*)) using the multiple threshold method, rejecting *D. popovi* and of *D. zivici* sp. nov. as different entities. GMYC analysis of a phylogenetic tree estimated using partial mt16S sequence data suggests 12 groups [6 morphological species] (aggregating overpartitioned (*D. bureschi* [partly] + *D. zivici* sp. nov. [partly]) twice, (*D. bureschi* [partly] + *D. discophoroides* [partly] + *D. dardanicus* [partly]), aggregating (*D. osogovicus* + *D. muranyorum*) and (*D*. *balcanicus* + *D. popovi*)) using the single threshold method, suggesting *D. popovi* differentiated from two clades comprising *D. zivici* sp. nov. partially — and 24 groups [6 morphological species] (aggregating overpartitioned (*D. bureschi* [partly] + *D. zivici* sp. nov. [partly]) twice, (*D. bureschi* [partly] + *D. dardanicus* [partly]) twice, (*D. osogovicus* [partly] + *D. muranyorum* [partly]) twice, aggregating (*M. austriacus* + *A. waringeri*); overpartitioning *D. osogovicus*, *D. muranyorum*, *D. zivici* sp. nov., *D. discophorus*, *E*. *dalecarlica*) using the multiple threshold method, suggesting *D. popovi* differentiated from a multitude of *D. zivici* sp. nov. entities. Results of GMYC analysis of a single gene phylogeny of partial nuCADH sequences suggest 3 groups [1 morphological species] (aggregating (*D. bureschi* + *D. discophoroides* + *D. osogovicus* + *D. dardanicus* + *D. muranyorum* + *D. balcanicus* + *D. zivici* sp. nov. + *D. popovi* + *D. discophorus* + *E. keroveci* + *E. dalcarlica*) and (*M. austriacus* + *A. waringeri*)) using the single threshold method, rejecting *D. popovi* and of *D. zivici* sp. nov. as different entities — and 9 groups [2 morphological species] (aggregating (*D. discophoroides* + *D. balcanicus*), (*D. osogovicus* + *D. dardanicus*), (*D. zivici* sp. nov. + *D. popovi*), (*E. keroveci* + *E. dalecarlica*), and (*D. discolor* + *M. melampophylax* + *A. waringeri*); overpartitioning *D. muranyorum*) using the multiple threshold method, rejecting *D. popovi* and of *D. zivici* sp. nov. as different entities. Results of GMYC analysis based on a single gene phylogeny of nuWnt1 suggest 11 groups [8 morphological species] (overpartitioning *D. muranyorum*) using both the single threshold method — and 10 groups [8 morphological species] (aggregating overpartitioned *D. muranyorum* + *D. discophorus*) using the mulitple threshold method, but is generally not informative regarding the status of *D. popovi* and *D. zivici* sp. nov. as data are missing; however, all specimens of *D*. *zivici* sp. nov. are recovered as a distinct group. GMYC analysis of single gene trees of partial nu28S sequence data suggest 3 groups (aggregating (*D. bureschi* + *D. discophoroides* + *D. osogovicus* + *D. dardanicus* + *D. balcanicus*), (*D. zivici* sp. nov. + *D. popovi* + *D. discophorus*) and (*D. muranyorum* + *E. keroveci* + *E. dalecarlica* + *D. discolor* + *M. melampophylax* + *A. waringeri*)) using the single threshold method, rejecting *D. popovi* and of *D. zivici* sp. nov. as different entities — and 18 groups [4 morphological species] (aggregating (*D. bureschi* [partly] + *D. osogovicus* [partly]), (*D. osogovicus* [partly] + *D. balcanicus*), (*D. popovi* [partly] + *D. zivici* sp. nov. [partly]), (*E. keroveci* [partly] + *E. dalecarlica*), and (*M. austriacus* + *A. waringeri*); overpartitioning *D. osogovicus, D. muranyorum*, *D. zivici* sp. nov., *D. popovi*, *E. keroveci*) using the multiple threshold method, rejecting *D. popovi* and of *D. zivici* sp. nov. as different entities.

#### Poisson tree process

Results from PTP based on a single gene phylogeny for mtCOI-5P suggests 15 groups [10 morphological species] (aggregating (*Drusus bureschi* + *D. discophoroides* + *D. osogovicus*); overpartitioning *D. zivici* sp. nov.), and suggests *D. popovi* differentiated from several *D. zivici* sp. nov. entities — results from bPTP suggest 20 groups [10 morphlogical species] (overpartitioning *D. bureschi*, *D. osogovicus*, *D*. *zivici* sp. nov.), and suggests *D. popovi* differentiated from several *D. zivici* sp. nov. entities. Results of PTP analysis based on a single gene genealogy of mtCOI-3P suggest 12 groups [9 morphological species] (aggregating (*Drusus bureschi* + *D. discophoroides* + *D. osogovicus* + *D. balcanicus*); overpartitioning *D. zivici* sp. nov.), and suggests *D. popovi* and *D. zivici* sp. nov. as different entities — bPTP suggests 17 groups [11 morphological species] (overpartitioning *D. bureschi*, *D. osogovicus*, *D*. *zivici* sp. nov.), and suggests *D. popovi* differentiated from several *D. zivici* sp. nov. entities. Based on a single gene phylogeny of partial mt16S genetic sequences, PTP suggests 15 groups [8 morphological species] (aggregating (*D. bureschi* [partly] + *D. zivici* sp. nov. [partly]) twice, (*D. bureschi* [partly] + *D. discophoroides* [partly] + *D. dardanicus* [partly]), and (*D. osogovicus* + *D. muranyorum*); overpartitioning *D. bureschi*, and *D. dardanicus*), and suggests *D. popovi* as differentiated from several clades containing *D. zivici* sp. nov. partially — using the same data, bPTP suggests 24 groups [7 morphological species] (overpartitioning *D. bureschi*, *D. osogovicus*, *D*. *muranyorum*, *D*. *zivici* sp. nov., *D. discophorus*, *E*. *dalecarlica*), and recovers *D. popovi* differentiated from several *D. zivici* sp. nov. entities. Poisson tree process analysis of a single gene tree of nuCADH suggests 16 groups [12 morphological species] (overpartitioning *D. muranyorum*, *D. discolor*) — bPTP recovers the same set of groups; both suggest *D. popovi* and *D. zivici* sp. nov. as different entities. PTP results based on a single gene tree of partial nuWnt1 sequence data suggest 16 groups [4 morphological species] (aggregating (*D*. *dardanicus* + *D. osogovicus*), overpartitioning *D. bureschi*, *D. muranyorum*, *D. zivici* sp. nov., and *E. keroveci*) — results of bPTP suggest 17 groups [3 morphological species] (aggregating (*D*. *dardanicus* + overpartitioned *D. osogovicus*), overpartitioning *D. bureschi*, *D. osogovicus*, *D. muranyorum*, *D. zivici* sp. nov., and *E. keroveci*), but is generally not informative regarding the status of *D. popovi* and *D. zivici* sp. nov. as data are missing; further, specimens of *D*. *zivici* sp. nov. are recovered in 2 distinct groups. Based on a single gene phylogeny of partial nu28S sequences, PTP identified 19 groups [6 morphological species] (aggregating (*D. bureschi* [partly] + *D. osogovicus* [partly]), (*D. zivici* sp. nov. + *D. popovi*), and (*E. keroveci* [partly] + *E. dalecarlica*); overpartitioning *D. bureschi*, *D. osogovicus, D. muranyorum*, *D. zivici* sp. nov., and *E. keroveci*), and rejects *D. popovi* and *D. zivici* sp. nov. as different entities — results of bPTP suggest 20 groups [6 morphological species] (aggregating (*D. bureschi* [partly] + *D. osogovicus* [partly]), (*D. zivici* sp. nov. + *D. popovi*), and (*E. keroveci* [partly] + *E. dalecarlica*); overpartitioning *D. bureschi*, *D. osogovicus, D. muranyorum*, *D. zivici* sp. nov., *D. popovi*, and *E. keroveci*), and rejects *D. popovi* and *D. zivici* sp. nov. as different entities.

#### Concatenated partial sequence data

Results from ABGD based on concatenated sequence data suggests 7 groups [6 morphological species] (aggregating *D. bureschi* + *D. discophoroides* + *D. osogovicus* + *D*. *dardanicus* + *D*. *muranyorum* + *D*. *balcanicus* + *D*. *zivici* sp. nov. + *D. popovi*), and rejects *D. popovi* and *D*. *zivici* sp. nov. as different entities.

#### Species tree analyses

Results from PTP based on a species tree estimated via *BEAST suggest 9 groups [8 morphological species] (aggregating (*D. bureschi* + *D. discophoroides* + *D. osogovicus* + *D. muranyorum* + *D. balcanicus*)), supporting *D. popovi* and *D. zivici* sp. nov. as different entities — bPTP recovers identical results. Species hypothesis estimation through single threshold GMYC on a species tree recovers 3 groups [2 morphological species] (aggregating (*D. bureschi* + *D. discophoroides* + *D. osogovicus* + *D*. *dardanicus* + *D*. *muranyorum* + *D*. *balcanicus* + *D*. *zivici* sp. nov. + *D. popovi* + *D. discophorus* + *E*. *keroveci* + *E*. *dalecarlica* + *D. discolor*)) — multiple threshold GMYC suggests 9 groups [8 morphological species] (aggregating (*D. bureschi* + *D. discophoroides* + *D. osogovicus* + *D. muranyorum* + *D. balcanicus*)), supporting *D. popovi* and *D. zivici* sp. nov. as different entities.

#### Combined species tree and species delimitation estimation

STACEY analysis suggests 14 groups equivalent to the 14 morphological species, and supports *D. popovi* and *D. zivici* sp. nov. as different entities using a collapse height of 0.0005 in species delimitation analysis (Fig. 1, Table 2).

### Effect of prior choice on STACEY delimitation results

Topology of final maximum clade credibility trees did not differ between runs while node support and branch lengths differed between single runs. STACEY recovered the same species hypotheses in runs with congruent ploidy settings, independent of growth rate and population scaling factor prior choice (Table 3). Ploidy settings considerably affected species delimitation results: When using the same ploidy settings for all loci, STACEY recovers 14 species hypotheses corresponding to the 14 morphological species included in the majority of analyses (Fig. 1., Tables 2 and 3).

**Table 3:**
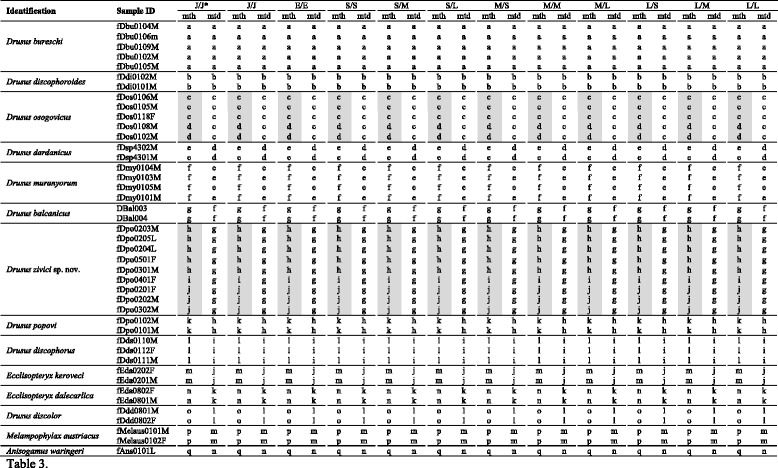
Tabular summary of results of molecular species delimitation via STACEY under different parameter settings

Specimens referred to by unique identifiers are grouped by species; results are displayed relative to morphologically distinguishable taxa and identical minuscules indicate group membership as inferred through STACEY using different prior settings. *Grey cells* indicate conflicts of species hypotheses and taxonomic benchmark, *white cells* indicate congruence of morphological delimitation and molecular species delimitation. Compound labels separated by slashes indicate prior settings for growth rate and population scale factor. Labels: J, Jeffreys prior; E/E, empiric priors corresponding to a logarithmic normal growth rate prior around (2.5, 1.2) and a logarithmic normal population scale factor prior around (−8.5, 2); S, logarithmic normal prior (M = 3, S = 1.5); M, logarithmic normal prior (M = 5, S = 2.5); L, logarithmic normal prior (M = 7, S = 3.5); mth, ploidy settings of mitochondrial loci corresponding to ¼ of the value used for nuclear loci; mtd, equal ploidy settings for all loci; asterisk, original analysis. For analytical details, see text. Order of specimens corresponds to order of specimens in Fig. 1

However, using one quarter of the nuclear ploidy value in STACEY analyses results in a higher number of species hypotheses (Table 3). These analyses concordantly suggest 17 groups [12 morphological species] (overpartitioning *D. osogovicus* and *D. zivici* sp. nov.) and support *D. popovi* and three distinct *D. zivici* sp. nov. groups as different entities using a collapse height corresponding to one tenth of the average branch length (oscillating between 0.0006 and 0.0007 between different analyses) in species delimitation analysis.

### Species description

*Drusus zivici* sp. nov. Kučinić, Previšić, Stojanović and Vitecek. http://zoobank.org/urn:lsid:zoobank.org:act:25366DC3-3829-41B0-8373-394D2A9DDFBA.

#### Material

Holotype. 1 male: Serbia, Stara Planina Mountains, spring of the river Tovarnička Reka; N43.3362367 E22.583983; 1493 m a.s.l.; 19.vi.2013–21.vi.2013; leg. M. Kučinić, K. Stojanović, M. Živić; specimen identifier fDpo0202M. Paratypes: 1 male, 1 female, 5 larvae: same data; specimen identifiers: 1 female: fDpo0201F, 1 male: fDpo0203M, 5 larvae: fDpo0204L–fDpo0208L. 2 males: Serbia, Stara Planina Mountains, spring of the river Rekička Reka; N43.372150 E22.625333; 1540 m a.s.l.; 19.vi.2013; leg. M. Kučinić, K. Stojanović, M. Živić; specimen identifiers: fDpo0301M, fDpo0302M. 1 female, 3 larvae: Serbia, Stara Planina Mountains, spring of Kaluđerske vode; N43.388690 E22.677934; 1930 m a.s.l.; 20.vi.2013; leg. M. Kučinić, K. Stojanović, M. Živić; specimen identifiers: 1 female: fDpo0401F, 3 larvae: fDpo0402L–fDpo0404L. 1 female: Serbia, Stara Planina Mountains, spring of the river Javorska Reka; N43.386150 E22.689817; 1890 m a.s.l.; 20.vi.2013; leg. M. Kučinić, K. Stojanović, M. Živić; specimen identifier: fDpo0501F.

Holotype and paratypes currently in coll. W. Graf, will be deposited in the Biologiezentrum des Oberösterreichischen Landesmuseums, Linz, Austria, and the Senckenberg Museum Frankfurt, Frankfurt am Main, Germany.

#### Type locality

Serbia, Stara Planina Mountains, Midžor Massiv.

#### Diagnosis

Males of the new species are most similar to *Drusus popovi* but exhibit (1) subcircular, elongate superior appendages in lateral view; (2) high tips of the intermediate appendages in lateral view, high and wide tips of intermediate appendages in caudal view; (3) suboval, elongate, approximately straight inferior appendages in lateral view; and (4) a high segment IX with a distinct, rounded, caudad medial protrusion in lateral view. *Drusus popovi* males have subcircular, short superior appendages; short and narrow tips of the intermediate appendages in lateral and caudal view; suboval, dorsadly curved inferior appendages; a wide segment IX lacking a distinct medial indentation in lateral view.

Females of the new species are most similar to females of *D. popovi* but exhibit (1) in dorsal view distinct, rounded lateral shoulders of segment X, (2) in dorsal view a ragged outline of the lateral lobes of segment X. *Drusus popovi* females have an evenly rounded lateral outline of segment X in dorsal view and evenly rounded lateral lobes of segment X.

Larvae of the new species are most similar to *D. serbicus* Marinković-Gospodnetić as larvae of both species have an intermittent lateral line ([98, 99]), but exhibit a pronotum with a distinct, rounded pronotal ridge (type B sensu [98]). Larvae of *D. serbicus* have an annular pronotal ridge (type E sensu [98]).

#### Description

##### Adults

Habitus fawn to yellow; head and thorax yellow, head with a dark mark around ocelli, metathorax with a dark mark, abdominal sclerites and tergites brown; cephalic and thoracic setal areas pale; cephalic, thoracic and abdominal setation blond; legs light yellow, distally darker; haustellum and intersegmental integument pale, whitish. Wings yellow to fawn, with blond setae. Male maxillary palp 3-segmented. Forewing length 7.9–10 mm, spur formula 1–3–3 in males; forewing length 8–10 mm, spur formula 1–3–3 in females.

##### Male genitalia (Fig. 2a-e)

Tergite VIII brown, in dorsal view cranially broadly incised; lacking setation; spinate area as two suboval laterocaudal lobes medially connected by a broad band of spines, embracing a medial wide indentation, flanked by membraneous less sclerotized areas. Ninth abdominal segment (IX) in caudal view ventrally as wide as dorsally; in lateral view medially with a distinct, rounded caudad protrusion and a ventral protrusion, embracing the base of the inferior appendages. Superior appendages in lateral view subcircular, dorsally elongate; in caudal and dorsal view compressed, medially concave. Intermediate appendages in lateral view rounded, rough, high (higher than the superior appendages); in dorsal view the tips medially more proximal, extending widely laterally: bar-shaped, laterally rounded, rough; in caudal view rectangular, tips as wide as the base of the intermediate appendages. Inferior appendages (gonopods sensu [62]) in lateral view subovate with a slight triangular dorsomedial protrusion, straight; in caudal, dorsal and ventral view proximal part broad, distal part slender, straight. Parameres simple, with a distinct medial thorn-like spine preceeded by 2 smaller spines.

**Fig. 2:**
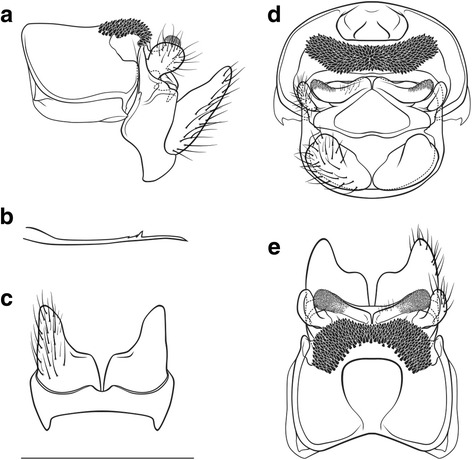
Male genitalia of *Drusus zivici* sp. nov. **a** *Left* lateral view. ** b** *Left* paramere, lateral view. **c** Ventral view. **d** Caudal view. **e** Dorsal view. Setation not shown on *right side*, scale bar indicates 1 mm. Del. Vitecek

##### Female genitalia (Fig. 3a-d)

Segment IX setation abundant, concentrated in the dorsal half; lateral lobe of segment IX membraneous, in lateral view oblique triangular, the ventral edge ventrally protruding and approximately twice as long as the dorsal edge, with a distinctly sclerotized setose dorsal part protruding caudally, in dorsal and ventral view slender, projecting laterally, in caudal view dorsal sclerotized setose part somewhat triangular. Segment X in lateral view oblique rectangular, with a distal, triangular caudal protrusion; in dorsal view hexagonal, medially widest, with rounded shoulders, 2 small median caudal lobes, and distally with 2 semi-circular, irregularly-edged lateral lobes, each with a lateral setose area; ventrally unsclerotized, open. Supragenital plate in lateral view quadrangular, ventrally longer than dorsally, with a small dorsal protrusion, caudal line slightly indent; in ventral view quadrangular with a medioventral indentation forming 2 rounded lateroventral lobes; in caudal view wide rectangular, dorsally slightly wider than ventrally, with a dorsomedial protrusion; in dorsal view with a medial dorsal protrusion. Vulvar scale in lateral view triangular, rather straight, approximately as long as the supragenital plate; in ventral view slender with 3 lobes: 2 lateral lobes, digitiform, roundly oval, straight; 1 median, digitiform, of lesser width than lateral lobes, length approximately 2/3rds of that of lateral lobes.

**Fig. 3:**
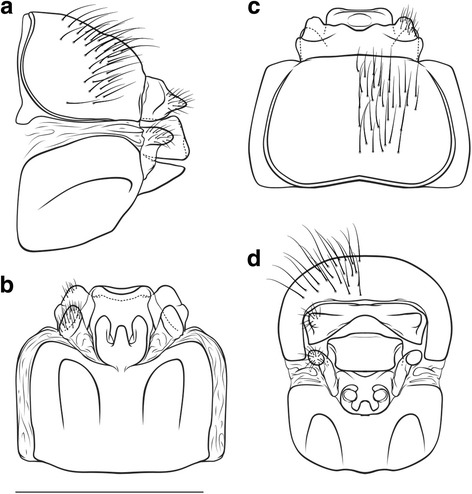
Female genitalia of *Drusus zivici* sp. nov. **a** *Left* lateral view. **b** Ventral view. **c** Dorsal view. **d** Caudal view. Setation not shown on left side, scale bar indicates 1 mm. Del. Vitecek

##### Fifth instar larva (Fig. 4a–j)

**Fig. 4:**
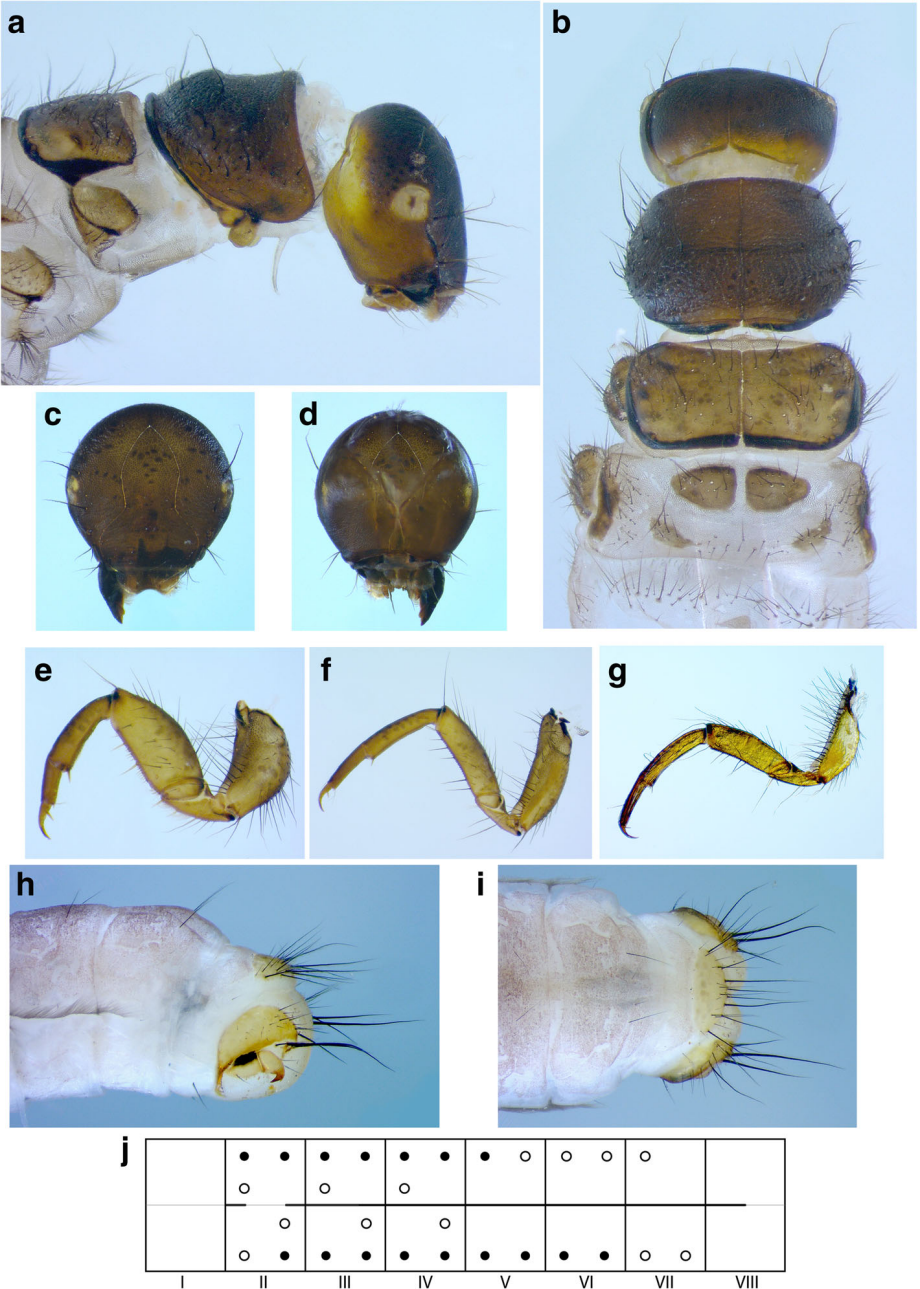
Larval characters of *Drusus zivici* sp. nov. **a** Larval head, prothorax and mesothorax, *right* lateral view. **b** Larval head, prothorax, mesothorax and metathorax, dorsal view. **c** Larval head, cranial view. **d** Larval head, caudal view. **e** Proleg, anterior view. **f** Mesoleg, anterior view. **g** Metaleg, anterior view. **h** Abdominal segments VII-X, lateral view. **i** Abdominal segments VII-X, dorsal view. **j** Schematic representation of the larval abdomen, depicting *lateral line* and abdominal gill arrangements. *Filled circles* indicate position of abdominal gills relative to full abdominal gill arrangement (*empty circles*)

Head capsule hypognathous, finely granulated, brown dorsally fading to fawn ventrally; 18 pairs of primary setae present: all brown with #1, 7, 9, 14 long and the rest shorter (Fig. 4c-d); antennae located on low carinae, each carina longer than high (Fig. 4a), both carinae curved mediad; mandibles toothless. Pronotum dark brown, coarsely granulated; distinct medial ridge present, rounded, steeper anteriorly in lateral view; recumbent white setae present, but scarce in the median third (Fig. 4a-b); pronotal horn present. Mesonotum completely covered by 2 sclerites, brown, with darker apodemes; edges black; *sa*1 comprising 4–10 setae, *sa*2 and *sa*3 connected, comprising 28–42 setae in total on each sclerite (Fig. 4b). Metanotum with 3 pairs of sclerites: anteriomedian sclerites subtriangularly ovoid, dark brown with 11–15 setae; posteromedian sclerites rhomboid, fawn, with 14–18 setae; lateral sclerites long, curved dorsally in lateral view, pale brown fading to yellow ventrally with a dark median spot and 20–28 setae (Fig. 4b). Legs brown, distally slightly lighter (Fig. 4e–g). Abdomen white (Fig. 4h), dorsal gills from II praesegmental position to V praesegmental position, lateral gills absent, ventral gills from II postsegmental position to VI postsegmental position; lateral line as a short segment in the first quarter of II, continuously from last quarter of II to first half of VIII (Fig. 4j); abdomen I with 1 dorsal and 2 lateral protuberances, posterior sclerites absent on lateral protuberances, setal areas *sa*1–3 fused dorsally and ventrally, sternum bearing 2 setae with distinct basal plates; abdomen VIII with 2 long and 2 short posterodorsal setae on either side; abdomen IX with 1 posterodorsal seta on either side, dorsal sclerite IX semicircular, pale brown with 7 long and several shorter setae (Fig. 4h-i). Case simple, constructed of mineral particles.

#### Etymology

Named for Miroslav Živić, biophysicist, for his continuous support of faunistic surveys.

## Discussion

### Drusinae diversity in the Balkans

Integrated assessment of morphology and results from multi-locus molecular species delimitation corroborate the new species *D. zivici* and indicate a sister species relationship with *D. popovi*. The description of this species increases total Drusinae diversity in the Balkans to 43 species [50–58, 64], demonstrating the significance of this region comprising a highly endemic fauna and flora for European biodiversity [100]. High diversity of aquatic taxa in the Balkans can be attributed to species communities of isolated habitats [101–104], including numerous Drusinae species. Intriguingly, Drusinae distribution patterns in the Balkans indicate continuance of segregated populations potentially dissociated by climatic or orogenetic processes that in combination with limited dispersal potential [52, 105] resulted in small-scale allopatric speciation and extant disjunct distribution of species. As several other taxonomic groups exhibit combinations of ecological traits similar to those of Drusinae (e.g., some Leuctridae, *Consorophylax* spp.; [15, 106–108]), Drusinae may serve as model taxon to estimate unexplored biodiversity. Indeed, recent taxonomic surveys recovered several new Drusinae species in the Western Balkans, demonstrating the necessity and urgency of organismic studies in Europe [109]. Additionally, the Balkan aquatic biodiversity is imminently threatened by anthropogenic habitat modification and climate change [110, 111]. Endemic taxa such as the majority of Drusinae species are particularly vulnerable to both climate change and habitat degradation [112–115]. Socio-economic change and the resulting acceleration of natural resource exploitation [110, 116–121] threaten natural habitats world-wide with deleterious effects on biodiversity.

Conservation measures to counter such development crucially depend on comprehensive faunistic data to identify the most critically imperiled habitats and implement adequate protection measures. While such comprehensive data is currently not available, the highly diversified and ecologically specialized Drusinae could serve as umbrella taxon for highland stream and spring habitats in the Western Balkans.

### Model taxon, limitations of the molecular dataset and molecular species delimitation

The Drusinae are morphologically well-defined, yet high diversity and intricate morphological characters make identification of several species challenging. Recognition and delineation of new species thus requires high taxonomic skill, and is best supported by additional, independent information such as molecular sequence data. We therefore assume that the Drusinae are representative of taxa that are likely to be investigated using molecular species delimitation tools. The molecular markers used in this study were successfully used to infer species-level relationships in Drusinae and were also found to corroborate taxonomic status of various new species within Drusinae [55, 56, 64, 68]. We thus assume they are sufficiently informative to support molecular taxon delimitation. Indeed, combined species tree estimation and taxa delimitation inferred on a multi-locus data set via STACEY delineated 14 entities corresponding to the 14 morphologically distinguishable species included.

However, the majority of molecular species delimitation tools exploiting single-locus data only did not produce conclusive taxon hypotheses when benchmarked against morphologically distinct entities. Likewise, automated barcode gap analyses on a concatenated molecular data set and GMYC and PTP analyses of a species tree estimated through *BEAST did not recover species hypotheses corresponding to morphologically identifiable species.

Taxonomic estimates based on different loci differ distinctly, indicating a certain necessity to select loci based on their informativeness [122]. We found delimitation results inferred on single locus data to reflect locus variability/proportion of parsimony-informative sites, as more variable loci led to a higher number of proposed species hypotheses. However, the majority of these delimitations are taxonomically not informative. We consequently suggest that, if only single locus data be used, a locus should be selected (ideally through benchmarking locus informativeness based on a known set of species-level taxa) that is conservative for single species in the focal group (e.g., [67]). Similar to our results, other studies report overpartitioning or overaggregation of morphological species by single-locus molecular taxa delimitation tools. Differential variability of molecular markers and differences in phylogenetic inference method used as well as effects of population sizes and speciation rates, but also low traditional taxonomic resolution were previously found to affect significance of molecular species delimitation [97, 122–125]. Altogether, single locus molecular species delimitation and identification tools currently seem limited in their general applicability. Results obtained here corroborate overaggregation and overpartitioning of morphological species by several single-locus molecular species delimitation tools, indicating a moderate potential as additional information source in integrative taxonomy [97, 122–125]. Ultimately, this problem is also related to deviation of single gene trees from the true species tree [126, 127]. STACEY likely outperforms single-locus approaches by directly integrating available information in a multispecies coalescent model to estimate a SMC tree. Further, we found the STACEY algorithm to be rather impervious to deviations in prior space controlling model speciation rate and population sizes. However, substantial variation of delimitation results can be induced when modifying ploidy settings. In line with [93–95] we argue that using the same ploidy settings for all loci represents a more robust approach to estimate species or minimal cluster trees by equally weighting variability in each locus. Our results strongly support promoting STACEY as the method of choice for integrative taxonomy, at least in caddisflies. Yet, further studies are necessary to gauge how well this method performs in other groups.

### Integrating molecular species delimitation and traditional taxonomy

Molecular species delimitation and identification tools can be used to infer operational taxon units for evolutionary or ecological analysis, or to obtain alternative taxa hypotheses or discover distinct evolutionary lineages in morphologically uniform taxa [128–134]. Using these tools is particularly beneficial for studying evolutionary or ecological patterns in taxonomically understudied areas [128, 134]. When integrated into larger databases these data on evolutionary lineages and species can be further used for large-scale evolutionary or phylogenetic studies [66]. Also, taxonomic resolution in ecological assessment of water quality could be greatly increased through molecular species identification [131, 135]. Such an approach would impart increased and standardized resolution of existing multi-metric indices and thus harbours the potential to enhance existing water resource management schemes.

However, while integrative taxonomy based on (initial) molecular genetic species identification has been proposed as remedy to the taxonomic impediment [136–138], the majority of taxonomic studies do not exploit this opportunity. This is likely due to financial or temporal limitations, and because species delimitation studies – including some of our own – do not follow through with formal species description or implementation of other taxonomic consequences.

Nevertheless, the potential to develop and test species hypotheses using molecular data is likely to act as an incentive for accelerated taxonomic and ecological research. Collaborative integrative taxonomic projects comprising molecular taxonomists and classical taxonomists are particularly likely to expedite discovery of global biodiversity. Increased collaborative taxonomic efforts will further provide a wealth of information for other disciplines of biological sciences, like biomonitoring of aquatic ecosystems [132, 133, 139–141], or conservation ecology [142–146]. Currently, constraints on integrative taxonomy, community meta-barcoding, or eDNA approaches in environmental monitoring assays are imposed by inadequate completeness and precision of molecular databases [19, 147–150]. We anticipate collaborative integrative taxonomic approaches will accelerate alpha-taxonomy and thus provide reference data in addition to on-going international efforts to develop reference libraries. Further, development of conservation management plans or novel tools for aquatic ecosystem assessments will benefit from data thus compiled.

## Conclusions

Only multi-locus molecular species delimitation via STACEY reliably delineated molecular species corresponding to morphologically identifiable taxa, confirming a priori expectations on taxonomic significance of different molecular species delimitation tools. We assume that taxonomically relevant molecular species delimitation tools hold potential to accelerate identification of new species (cf. [137, 138]), local and global biodiversity estimation and thus enforcement of conservation policies [151] by providing a meaningful assessment of biodiversity richness. However, the capacities of purely molecular species identification based on single-locus data seem to have been overestimated. This clearly demonstrates that while molecular genetic procedures will likely be of relevance in routine monitoring applications, they are currently not fit to serve as surrogate for ‘classical’ explorative taxonomy [152–154].

Nevertheless, we expect multispecies-coalescent-based molecular species delimitation to mitigate the taxonomic impediment and accelerate taxonomic and ecological studies. Under the prevailing biodiversity crisis we direly need to uncover what we are losing fastest – life’s uncharted diversity [150, 154]. Thus, we advocate a truly holistic integrative taxonomy in order to comprehensively scrutinize our planets declining biodiversity, and so provide essential information for applied biologists, ecologists, conservationists and policy-makers.

## Additional file

Additional file 1: Specimen and data set details. Description of data: Summary of collection details, molecular sequence data used and voucher specimen storage. Abbreviations: Lat, latitude; Lon, longitude. (PDF 415 kb)

## References

[1] Sites JW, Marshall JC (2004). Operational criteria for delimiting species. Annu Rev Ecol.

[2] Dayrat B (2005). Towards integrative taxonomy. Biol J Linn Soc.

[3] Schlick-Steiner BC, Steiner FM, Seifert B, Stauffer C, Christian E, Crozier RH (2010). Integrative taxonomy: a multisource approach to exploring biodiversity. Annu Rev Entomol.

[4] Barbour MT, Gerritsen J, Snyder BD, Stribling JB: Rapid bioassessment protocols for use in wadeable streams and rivers: periphyton, benthic macroinvertebrates and fish*.* 2nd edition. Washington: USEPA; 1999.

[5] Barbour MT, Yoder CO: The multimetric approach to bioassessment, as used in the United States of America. In Wright JF, Sutcliffe DW, Furse MT. Ambleside, editors. Assessing the biological quality of fresh waters. Ambleside: Freshwater Biological Association; 2000:281–92.

[6] Yasuoka J, Levins R (2007). Ecology of vector mosquitoes in Sri Lanka--suggestions for future mosquito control in rice ecosystems. Southeast Asian J Trop Med Public Health.

[7] Perrin P, Herbreteau V, Hugot JP, Morand S: Biogeography, humans, and their parasites. In Morand S, Krasnov BR, editors. The biogeography of host-parasite interactions. New York: Oxford University Press; p. 41–58.

[8] Godfray HCJ (2013). Mosquito ecology and control of malaria. J Anim Ecol.

[9] Osório HC, Zé-Zé L, Amaro F, Nunes A, Alves MJ (2013). Sympatric occurrence of *Culex pipiens* (Diptera, Culicidae) biotypes *pipiens*, *molestus* and their hybrids in Portugal, Western Europe: feeding patterns and habitat determinants. Med Vet Entomol.

[10] Emery NJ (2014). Enhanced species distribution models: a case study using essential population data from *Actinotus helianthi* (flannel flower).

[11] Harris DB, Gregory SD, Brook BW, Ritchie EG, Croft DB, Coulson G (2014). The influence of non-climate predictors at local and landscape resolutions depends on the autecology of the species. Austral Ecol.

[12] Emery NJ, Henwood MJ, Offord CA, Wardle GM (2015). *Actinotus helianthi* populations across a wide geographic range exhibit different climatic envelopes and complex relationships with plant traits. Int J Plant Sci.

[13] Börstler J, Jöst H, Garms R, Krüger A, Tannich E, Becker N, et al. Host-feeding patterns of mosquito species in Germany. Parasit Vectors. 2016;9:1–14. 10.1186/s13071-016-1597-z PMC4893232 27259984

[14] Schmidt-Kloiber A, Hering D (2015). www.freshwaterecology.info – an online tool that unifies, standardises and codifies more than 20,000 European freshwater organisms and their ecological preferences. Ecol Indic.

[15] Graf W, Murphy J, Zamora-Muñoz C, Jesus Lopez-Rodriguez M (2008). Distribution and ecological preferences of European freshwater organisms. Volume 1 - Trichoptera.

[16] Lenat DR, Resh VH (2001). Taxonomy and stream ecology - the benefits of genus- and species-level identifications. J N Am Benthol Soc.

[17] Bonada N, Prat N, Resh VH, Statzner B (2006). Developments in aquatic insect biomonitoring: a comparative analysis of recent approaches. Annu Rev Entomol.

[18] Waringer J, Graf W, Malicky H (2013). Problems associated with extrapolating ecological traits to higher-than-species level exemplified in the description of the larvae of *Potamophylax haidukorum* Malicky, 1999, *Potamophylax winneguthi* (Klapálek, 1902) and *Melampophylax austriacus* Malicky, 1990. Limnologica.

[19] Wheeler QD, Raven PH, Wilson EO (2004). Taxonomy: impediment or expedient?. Science.

[20] Boero F (2010). The study of species in the era of biodiversity: a tale of stupidity. Diversity.

[21] Ebach MC, Valdecasas AG, Wheeler QD (2011). Impediments to taxonomy and users of taxonomy: accessibility and impact evaluation. Cladistics.

[22] Steyskal GC (1967). Another view of the future of taxonomy. Syst Biol.

[23] Wheeler QD, Meier R (2000). Species concepts and phylogenetic theory: a debate.

[24] Will KW, Rubinoff D (2004). Myth of the molecule: DNA barcodes for species cannot replace morphology for identification and classification. Cladistics.

[25] Dubois A (2011). Species and “strange species” in zoology: do we need a ‘unified concept of species’?. Comptes Rendus Palevol.

[26] Will KW, Mishler BD, Wheeler QD (2005). The perils of DNA barcoding and the need for integrative taxonomy. Syst Biol.

[27] Valdecasas AG, Williams D, Wheeler QD (2008). “Integrative taxonomy” then and now: a response to Dayrat (2005). Biol J Linn Soc.

[28] Tautz D, Arctander P, Minelli A, Thomas RH, Vogler AP (2002). DNA points the way ahead of taxonomy. Nature.

[29] Tautz D, Arctander P, Minelli A, Thomas RH, Vogler AP (2003). A plea for DNA taxonomy. Trends Ecol Evol.

[30] Hebert PDN, Ratnasingham S, de Waard JR (2003). Barcoding animal life: cytochrome c oxidase subunit 1 divergences among closely related species. Proc Biol Sci.

[31] Wiens JJ (2007). Species delimitation: new approaches for discovering diversity. Syst Biol.

[32] O'Meara BC (2010). New heuristic methods for joint species delimitation and species tree inference. Syst Biol.

[33] Rannala B (2015). The art and science of species delimitation. Curr Zool.

[34] Leaché AD, Fujita MK (2010). Bayesian species delimitation in West African forest geckos (*Hemidactylus fasciatus*). Proc R Soc Lond B.

[35] Kubatko LS, Gibbs HL, Bloomquist EW (2011). Inferring species-level phylogenies and taxonomic distinctiveness using multilocus data in *Sistrurus* rattlesnakes. Syst Biol.

[36] Astrin JJ, Stüben PE, Misof B, Wägele JW, Gimnich F, Raupach MJ (2012). Exploring diversity in cryptorhynchine weevils (Coleoptera) using distance-, character- and tree-based species delineation. Mol Phylog Evol.

[37] Bannikova AA, Zemlemerova ED, Colangelo P, Sözen M, Sevindik M, Kidov AA (2015). An underground burst of diversity - a new look at the phylogeny and taxonomy of the genus *Talpa* Linnaeus, 1758 (Mammalia: Talpidae) as revealed by nuclear and mitochondrial genes. Zool J Linnean Soc.

[38] Lang AS, Bocksberger G, Stech M (2015). Phylogeny and species delimitations in European *Dicranum* (Dicranaceae, Bryophyta) inferred from nuclear and plastid DNA. Mol Phylog Evol.

[39] Mrinalini, Thorpe RS, Creer S, Lallias D, Dawnay L, Stuart BL, et al. Convergence of multiple markers and analysis methods defines the genetic distinctiveness of cryptic pitvipers. Mol Phylog Evol. 2015;92(C):266–79. 10.1016/j.ympev.2015.06.001 26162672

[40] Wu Y, Murphy RW (2015). Concordant species delimitation from multiple independent evidence: a case study with the *Pachytriton brevipes* complex (Caudata: Salamandridae). Mol Phylog Evol.

[41] Butcher BA, Smith MA, Sharkey MJ, Quicke DLJ. A turbo-taxonomic study of Thai *Aleiodes* (*Aleiodes*) and Aleiodes (*Arcaleiodes*) (Hymenoptera: Braconidae: Rogadinae) based largely on COI barcoded specimens, with rapid descriptions of 179 new species. Zootaxa. 2012;3457:1–232.

[42] Ceccarelli FS, Sharkey MJ, Zaldívar-Riverón A (2012). Species identification in the taxonomically neglected, highly diverse, neotropical parasitoid wasp genus *Notiospathius* (Braconidae: Doryctinae) based on an integrative molecular and morphological approach. Mol Phylog Evol.

[43] Riedel A, Sagata K, Surbakti S, Tänzler R, Balke M (2013). One hundred and one new species of *Trigonopterus* weevils from New Guinea. ZooKeys.

[44] Riedel A, Tänzler R, Balke M, Rahmadi C, Suhardjono YR. Ninety-eight new species of *Trigonopterus* weevils from Sundaland and the Lesser Sunda Islands. ZooKeys. 2014;467:1–162. 10.3897/zookeys.467.8206 PMC4296478 25610340

[45] Pante E, Schoelinck C, Puillandre N (2014). From integrative taxonomy to species description: one step beyond. Syst Biol.

[46] Steiner FM, Pautasso M, Zettel H, Moder K, Arthofer W, Schlick-Steiner BC (2015). A falsification of the citation impediment in the taxonomic literature. Syst Biol.

[47] Pauls SU, Blahnik RJ, Zhou X, Wardwell CT, Holzenthal RW (2010). DNA barcode data confirm new species and reveal cryptic diversity in Chilean *Smicridea (Smicridea)* (Trichoptera: Hydropsychidae). J N Am Benthol Soc.

[48] Pauls SU, Lumbsch HT, Haase P (2006). Phylogeography of the montane caddisfly *Drusus discolor*: evidence for multiple refugia and periglacial survival. Mol Ecol.

[49] Pauls SU, Theissinger K, Ujvarosi L, Bálint M, Haase P (2009). Patterns of population structure in two closely related, partially sympatric caddisflies in Eastern Europe: historic introgression, limited dispersal, and cryptic diversity. J N Am Benthol Soc.

[50] Previšić A, Graf W, Vitecek S, Kučinić M, Bálint M, Keresztes L (2014). Cryptic diversity of caddisflies in the Balkans: the curious case of *Ecclisopteryx* species (Trichoptera: Limnephilidae). Arthropod Sys Phylogeny.

[51] Previšić A, Schnitzler J, Kučinić M, Graf W, Ibrahimi H, Kerovec M (2014). Microscale vicariance and diversification of Western Balkan caddisflies linked to karstification. Freshw Sci.

[52] Geismar J, Haase P, Nowak C, Sauer J, Pauls SU (2015). Local population genetic structure of the montane caddisfly *Drusus discolor* is driven by overland dispersal and spatial scaling. Freshw Biol.

[53] Marinković-Gospodnetić M, Malicky H (1974). The differentiation of Drusus species of the group bosnicus. Proceedings of the First International Symposium on Trichoptera.

[54] Malicky H (2004). Atlas of European Trichoptera.

[55] Vitecek S, Kučinić M, Oláh J, Previšić A, Bálint M, Keresztes L (2015). Description of two new filtering carnivore *Drusus* species (Limnephilidae, Drusinae) from the Western Balkans. ZooKeys.

[56] Vitecek S, Previšić A, Kučinić M, Bálint M, Keresztes L, Waringer J (2015). Description of a new species of *Wormaldia* from Sardinia and a new *Drusus* species from the Western Balkans (Trichoptera, Philopotamidae, Limnephilidae). ZooKeys.

[57] Ibrahimi H, Vitecek S, Previšić A, Kučinić M, Waringer J, Graf W (2016). *Drusus sharrensis* sp. n. (Trichoptera, Limnephilidae), a new species from Sharr National Park in Kosovo, with molecular and ecological notes. ZooKeys.

[58] Ibrahimi H, Kučinić M, Vitecek S, Waringer J, Graf W, Previšić A (2015). New records for the Kosovo caddisfly fauna with the description of a new species, *Drusus dardanicus* sp. nov. (Trichoptera: Limnephilidae). Zootaxa.

[59] Previšić A, Walton C, Kučinić M, Mitrikeski PT, Kerovec M (2009). Pleistocene divergence of Dinaric *Drusus* endemics (Trichoptera, Limnephilidae) in multiple microrefugia within the Balkan peninsula. Mol Ecol.

[60] Böhm A, Bartel D, Szucsich NU, Pass G (2011). Confocal imaging of the exo- and endoskeleton of Protura after non-destructive DNA extraction. Soil Organisms.

[61] Nielsen A (1957). A comparative study of the genital segments and their appendages in male trichoptera. Biol Skr Kong Dansk Vid Sel.

[62] Snodgrass RE (1935). Principles of insect morphology.

[63] Thomson RE, Holzenthal RW (2010). New Neotropical species of the genus *Austrotinodes* Schmid (Trichoptera: Ecnomidae). Zootaxa.

[64] Vitecek S, Graf W, Previšić A, Kučinić M, Oláh J, Bálint M (2015). A hairy case: the evolution of filtering carnivorous Drusinae (Limnephilidae, Trichoptera). Mol Phylog Evol.

[65] Malm T, Johanson KA, Wahlberg N (2013). The evolutionary history of Trichoptera (Insecta): a case of successful adaptation to life in freshwater. Syst Ent.

[66] Zhou X, Frandsen PB, Holzenthal RW, Beet CR, Bennett KR, Blahnik RJ (2016). **The Trichoptera barcode initiative: a strategy for generating a species-level tree of life**. Philos Trans R Soc B Biol Sci.

[67] Zhou X, Kjer KM, Morse JC (2007). Associating larvae and adults of Chinese Hydropsychidae caddisflies (Insecta:Trichoptera) using DNA sequences. J N Am Benthol Soc.

[68] Pauls SU, Graf W, Haase P, Lumbsch HT, Waringer J (2008). Grazers, shredders and filtering carnivores—the evolution of feeding ecology in Drusinae (Trichoptera: Limnephilidae): insights from a molecular phylogeny. Mol Phylog Evol.

[69] Kearse M, Moir R, Wilson A, Stones-Havas S, Cheung M, Sturrock S (2012). Geneious basic: an integrated and extendable desktop software platform for the organization and analysis of sequence data. Bioinformatics.

[70] Katoh K, Standley DM (2013). MAFFT multiple sequence alignment software version 7: improvements in performance and usability. Mol Biol Evol.

[71] Tamura K, Stecher G, Peterson D, Filipski A, Kumar S (2013). MEGA6: molecular evolutionary genetics analysis version 6.0. Mol Biol Evol.

[72] Bouckaert R, Heled J, Kühnert D, Vaughan T, Wu C-H, Xie D (2014). BEAST 2: a software platform for Bayesian evolutionary analysis. PLoS Comput Biol.

[73] Rambaut A, Suchard MA, Xie D, Drummond AJ. Tracer v1.6. http://beast.bio.ed.ac.uk/Tracer. Accessed 12 Feb 2014.

[74] Drummond AJ, Suchard MA, Xie D, Rambaut A (2012). Bayesian phylogenetics with BEAUti and the BEAST 1.7. Mol Biol Evol.

[75] Heled J, Drummond AJ (2010). Bayesian inference of species trees from multilocus data. Mol Biol Evol.

[76] Zhang J, Kapli P, Pavlidis P, Stamatakis A (2013). A general species delimitation method with applications to phylogenetic placements. Bioinformatics.

[77] O'Meara BC, Ané C, Sanderson MJ, Wainwright PC (2006). Testing for different rates of continuous trait evolution using likelihood. Evolution.

[78] Kubatko LS, Carstens BC, Knowles LL (2009). STEM: species tree estimation using maximum likelihood for gene trees under coalescence. Bioinformatics.

[79] Kubatko LS (2009). Identifying hybridization events in the presence of coalescence via model selection. Syst Biol.

[80] Ence DD, Carstens BC (2011). SpedeSTEM: a rapid and accurate method for species delimitation. Mol Ecol Resour.

[81] Puillandre N, Lambert A, Brouillet S, Achaz G (2012). ABGD, Automatic Barcode Gap Discovery for primary species delimitation. Mol Ecol.

[82] Jukes TH, Cantor CR, Munro HN (1969). Evolution of protein molecules. Mammalian protein metabolism III.

[83] Kimura M (1980). A simple method for estimating evolutionary rates of base substitutions through comparative studies of nucleotide sequences. J Mol Evol.

[84] Kekkonen M, Hebert PDN (2014). DNA barcode-based delineation of putative species: efficient start for taxonomic workflows. Mol Ecol Resour.

[85] Kekkonen M, Mutanen M, Kaila L, Nieminen M, Hebert PDN (2015). Delineating species with DNA barcodes: a case of taxon dependent method performance in moths. PLoS One.

[86] Fujisawa T, Barraclough TG (2013). Delimiting species using single-locus data and the generalized mixed yule coalescent approach: a revised method and evaluation on simulated data sets. Syst Biol.

[87] Pons J, Barraclough T, Gomez-Zurita J, Cardoso A, Duran D, Hazell S (2006). Sequence-based species delimitation for the DNA taxonomy of undescribed insects. Syst Biol.

[88] Ezard T, Fujisawa T, Barraclough TG (2014). Splits: SPecies’ LImits by Threshold Statistics.

[89] R Core Team (2015). R: a language and environment for statistical computing.

[90] Jones G (2017). Algorithmic improvements to species delimitation and phylogeny estimation under the multispecies coalescent. J Math Biol.

[91] Jones G, Aydin Z, Oxelman B (2015). DISSECT: an assignment-free Bayesian discovery method for species delimitation under the multispecies coalescent. Bioinformatics.

[92] Drummond AJ, Bouckaert RR (2015). Bayesian evolutionary analysis with BEAST.

[93] Jacobsen F, Omland KE (2011). Species tree inference in a recent radiation of orioles (Genus Icterus): multiple markers and methods reveal cytonuclear discordance in the northern oriole group. Mol Phylog Evol.

[94] DuBay SG, Witt CC (2012). An improved phylogeny of the Andean tit-tyrants (Aves, Tyrannidae): more characters trump sophisticated analyses. Mol Phylog Evol.

[95] Jockusch EL, Martinez-Solano I, Timpe EK (2014). The effects of inference method, population sampling, and gene sampling on species tree inferences: an empirical study in slender salamanders (Plethodontidae: Batrachoseps). Syst Biol.

[96] Jones G. speciesDA.jar. http://www.indriid.com/2014/speciesDA.jar. Accessed 18 June 2015.

[97] Tang CQ, Humphreys AM, Fontaneto D, Barraclough TG, Paradis E (2014). Effects of phylogenetic reconstruction method on the robustness of species delimitation using single-locus data. Methods Ecol Evol.

[98] Waringer J, Previšić A, Kučinić M, Graf W, Vitecek S, Keresztes L, et al. Larval morphology of the Western Balkans endemic caddisflies *Drusus krusniki* Malicky 1981, *D. vernonensis* Malicky 1989, and *D. vespertinus* Marinković 1976 (Trichoptera, Limnephilidae, Drusinae). Zootaxa. 2016;4083:483–500. 10.11646/zootaxa.4083.4.2 PMC4789510 26985141

[99] Waringer J, Graf W, Bálint M, Kučinić M, Pauls SU, Previšić A, et al. Larval morphology and phylogenetic position of *Drusus balcanicus*, *D. botosaneanui*, *D. serbicus* and *D. tenellus* (Trichoptera: Limnephilidae: Drusinae). Eur J Entomol. 2015;112:344–61. 10.14411/eje.2015.037 PMC4793628 26997882

[100] Griffiths HI, Kryštufek B, Reed JM (2004). Balkan biodiversity.

[101] Petkovski T, Scharf BW, Keyser D. Freshwater Ostracoda (Crustacea) collected from caves and the interstitial habitat in Herzegovina, NW Balkan, with the description of two new species. Bull Soc Nat Luxemb. 2009;110:173–82.

[102] Wilke T, Schultheiß R, Albrecht C, Bornmann N, Trajanovski S, Kevrekidis T. Native *Dreissena* freshwater mussels in the Balkans: in and out of ancient lakes. Biogeosciences. 2010;7:3051–65.

[103] Pešić V, Glöer P (2013). A new freshwater snail genus (Hydrobiidae, Gastropoda) from Montenegro, with a discussion on gastropod diversity and endemism in Skadar Lake. ZK.

[104] Ivković M, Plant A (2015). Aquatic insects in the Dinarides: identifying hotspots of endemism and species richness shaped by geological and hydrological history using Empididae (Diptera). Insect Conserv Divers.

[105] Müller-Peddinghaus E, Hering D (2013). The wing morphology of limnephilid caddisflies in relation to their habitat preferences. Freshw Biol.

[106] Graf W, Vitecek S (2016). A new species of Limnephilidae (Insecta: Trichoptera) from the Western Alps (Insecta: Trichoptera). Zootaxa.

[107] Graf W, Vitecek S, Previšić A, Malicky H (2015). New species of Limnephilidae (Insecta: Trichoptera) from Europe: Alps and Pyrenees as harbours of unknown biodiversity. Zootaxa.

[108] Graf W, Lorenz AW, Tierno de Figueroa JM, Lücke S, Jesus Lopez-Rodriguez M, Davies C (2009). Distribution and ecological preferences of European freshwater organisms. Volume 2 - Plecoptera.

[109] Fontaine B, van Achterberg K, Alonso-Zarazaga MA, Araujo R, Asche M, Aspöck H, et al. New species in the old world: Europe as a frontier in biodiversity exploration, a test bed for 21st century taxonomy. PLoS One. 2012;7:e36881–7. 10.1371/journal.pone.0036881 PMC3359328 22649502

[110] Schwarz U (2015). Hydropower projects on the Balkan Rivers – update.

[111] Schwarz U (2012). Balkan Rivers – the blue heart of Europe.

[112] Hering D, Schmidt-Kloiber A, Murphy J, Lücke S, Zamora-Muñoz C, Jesus Lopez-Rodriguez M (2009). Potential impact of climate change on aquatic insects: a sensitivity analysis for European caddisflies (Trichoptera) based on distribution patterns and ecological preferences. Aquat Sci.

[113] de Figueroa JMT, López-Rodríguez MJ, Lorenz A, Graf W, Schmidt-Kloiber A, Hering D (2010). Vulnerable taxa of European Plecoptera (Insecta) in the context of climate change. Biodivers Conserv.

[114] Bálint M, Domisch S, Engelhardt CHM, Haase P, Lehrian S, Sauer J, et al. Cryptic biodiversity loss linked to global climate change. Nat Clim Chang. 2011;1:313–8.

[115] Conti L, Schmidt-Kloiber A, Grenouillet G, Graf W (2014). A trait-based approach to assess the vulnerability of European aquatic insects to climate change. Hydrobiologia.

[116] Foster GN: Conserving insects of aquatic and wetland habitats, with special reference to beetles. In Collins NM, Thomas JA, editors. The conservation of insects and their habitats. London; 1991:237–262.

[117] Polhemus DA (1993). Conservation of aquatic insects: worldwide crisis or localized threats?. Am Zool.

[118] Dudgeon D, Arthington AH, Gessner MO, Kawabata Z-I, Knowler DJ, Lévêque C (2006). Freshwater biodiversity: importance, threats, status and conservation challenges. Biol Rev.

[119] Barquin J, Death RG (2004). Patterns of invertebrate diversity in streams and freshwater springs in Northern Spain. Arch Hydrobiol.

[120] Vidic RD, Brantley SL, Vandenbossche JM, Yoxtheimer D, Abad JD (2013). Impact of shale gas development on regional water quality. Science.

[121] Zarfl C, Lumsdon AE, Berlekamp J, Tydecks L, Tockner K (2014). A global boom in hydropower dam construction. Aquat Sci.

[122] Olave M, Sola E, Knowles LL (2014). Upstream analyses create problems with DNA-based species delimitation. Syst Biol.

[123] Talavera G, Dincă V, Vila R (2013). Factors affecting species delimitations with the GMYC model: insights from a butterfly survey. Methods Ecol Evol.

[124] Dellicour S, Flot J-F (2015). Delimiting species-poor data sets using single molecular markers: a study of barcode gaps, Haplowebs and GMYC. Syst Biol.

[125] Schwarzfeld MD, Sperling FAH (2015). Comparison of five methods for delimitating species in *Ophion* Fabricius, a diverse genus of parasitoid wasps (Hymenoptera, Ichneumonidae). Mol Phylog Evol.

[126] Pamilo P, Nei M (1988). Relationships between gene trees and species trees. Mol Biol Evol.

[127] Maddison WP (1997). Gene trees in species trees. Syst Biol.

[128] Craft KJ, Pauls SU, Darrow K, Miller SE, Hebert PDN, Helgen LE, et al. Population genetics of ecological communities with DNA barcodes: an example from New Guinea Lepidoptera. Proc Natl Acad Sci. 2010;107:5041–6. 10.1073/pnas.0913084107 PMC2841870 20202924

[129] Keith R, Hedin M (2012). Extreme mitochondrial population subdivision in southern Appalachian paleoendemic spiders (Araneae: Hypochilidae: *Hypochilus*), with implications for species delimitation. J Arachnol.

[130] Parmakelis A, Kotsakiozi P, Stathi I, Poulikarakou S, Fet V. Hidden diversity of *Euscorpius* (Scorpiones: Euscorpiidae) in Greece revealed by multilocus species-delimitation approaches. Biol J Linn Soc. 2013;110:728–48.

[131] Pauls SU, Alp M, Bálint M, Bernabò P, Ciampor FJ, Čiamporová-Zaťovičová Z (2014). Integrating molecular tools into freshwater ecology: developments and opportunities. Freshw Biol.

[132] Stein ED, Martinez MC, Stiles S, Miller PE, Zakharov EV (2014). Is DNA barcoding actually cheaper and faster than traditional morphological methods: results from a survey of freshwater bioassessment efforts in the United States?. PLoS One.

[133] Zimmermann J, Glöckner G, Jahn R, Enke N, Gemeinholzer B (2014). Metabarcoding vs. morphological identification to assess diatom diversity in environmental studies. Mol Ecol Resour.

[134] Hoppeler F, Tachamo Shah RD, Shah DN, Jähnig SC, Tonkin JD, Sharma S (2016). Environmental and spatial characterisation of an unknown fauna using DNA sequencing - an example with Himalayan Hydropsychidae (Insecta: Trichoptera). Freshw Biol.

[135] Leese F, Altermatt F, Bouchez A, Ekrem T, Hering D, Meissner K (2016). DNAqua-net: developing new genetic tools for bioassessment and monitoring of aquatic ecosystems in Europe. RIO.

[136] Godfray HCJ (2007). Linnaeus in the information age. Nature.

[137] Tänzler R, Sagata K, Surbakti S, Balke M, Riedel A (2012). DNA barcoding for community ecology-how to tackle a hyperdiverse, mostly undescribed Melanesian fauna. PLoS One.

[138] Hubert N, Hanner R (2015). DNA barcoding, species delineation and taxonomy: a historical perspective. DNA Barcodes.

[139] Stein ED, White BP, Mazor RD, Jackson JK, Battle JM, Miller PE (2014). Does DNA barcoding improve performance of traditional stream bioassessment metrics?. Freshw Sci.

[140] Elbrecht V, Leese F (2015). Can DNA-based ecosystem assessments quantify species abundance? Testing primer bias and biomass-sequence relationships with an innovative metabarcoding protocol. PLoS One.

[141] Valentini A, Taberlet P, Miaud C, Civade R, Herder J, Thomsen PF (2016). Next-generation monitoring of aquatic biodiversity using environmental DNA metabarcoding. Mol Ecol.

[142] Moritz C (1994). Applications of mitochondrial DNA analysis in conservation: a critical review. Mol Ecol.

[143] Haig SM (1998). Molecular contributions to conservation. Ecology.

[144] Bickford D, Lohman DJ, Sodhi NS, Ng PKL, Meier R, Winker K (2007). Cryptic species as a window on diversity and conservation. Trends Ecol Evol.

[145] Haig SM, Bronaugh WM, Crowhurst RS, D'Elia J, Eagles-Smith CA, Epps CW, et al. Genetic applications in avian conservation. Auk. 2011;128:205–29.

[146] Bock F, Fennessy J, Bidon T, Tutchings A, Marais A, Deacon F (2014). Mitochondrial sequences reveal a clear separation between Angolan and South African giraffe along a cryptic rift valley. BMC Evol Biol.

[147] Harris DJ (2003). Can you bank on GenBank?. Trends Ecol Evol.

[148] Nilsson RH, Ryberg M, Kristiansson E, Abarenkov K, Larsson K-H, Kõljalg U (2006). Taxonomic reliability of DNA sequences in public sequence databases: a fungal perspective. PLoS One.

[149] Boykin LM, Armstrong K, Kubatko L, De Barro P (2012). DNA barcoding invasive insects: database roadblocks. Invertebr Syst.

[150] Collins RA, Cruickshank RH (2012). The seven deadly sins of DNA barcoding. Mol Ecol Resour.

[151] Cardoso P, Erwin TL, Borges PAV, New TR (2011). The seven impediments in invertebrate conservation and how to overcome them. Biol Conserv.

[152] Sluys R (2013). The unappreciated, fundamentally analytical nature of taxonomy and the implications for the inventory of biodiversity. Biodivers Conserv.

[153] Solís-Lemus C, Knowles LL, Ané C (2015). Bayesian species delimitation combining multiple genes and traits in a unified framework. Evolution.

[154] Dijkstra K-DB (2016). Restore our sense of species. Nature.

