# Supplementary material for: Integrative taxonomy by molecular species delimitation: multi-locus data corroborate a new species of Balkan Drusinae micro-endemics
Source: BMC Evol Biol. 2017 Jun 6;17:129. doi: 10.1186/s12862-017-0972-5 (PMC5461746; doi:10.1186/s12862-017-0972-5)
Supplement: Additional file 1: — Specimen and data set details. Description of data: Summary of collection details, molecular sequence data used and voucher specimen storage. Abbreviations: Lat, latitude; Lon, longitude. (PDF 415 kb) [file 12862_2017_972_MOESM1_ESM.pdf]

Process ID	Sample ID	Identification	Collection information	Lat	Lon	mtCOI-5P	mtCOI-3P	mt16S	nuCADH	nuWnt1	nu28S	Storing Institution
SPDRU124-14	DBal003	*Drusus balcanicus*	Bulgaria, Plovdiv, Karnare, Hristo Danovo; leg. Balint, Neu; 12-Jun-2008	42,7734	24,6186	658[0n]	541[0n]	362[0n]	846[1n]	346[0n]	1038[0n]	Senckenberg Forschungsinstitut und Naturmuseum
SPDRU125-14	DBal004	*Drusus balcanicus*	Bulgaria, Plovdiv, Karnare, Hristo Danovo; leg. Balint, Neu; 12-Jun-2008	42,7734	24,6186	658[0n]	541[0n]	362[0n]	847[1n]	346[0n]	1038[0n]	Senckenberg Forschungsinstitut und Naturmuseum
SPDRU147-14	fAns0101L	*Anisogamus waringeri*	France; leg. Graf; 09-Jun-2013			658[0n]	541[0n]	360[0n]	848[0n]	n.a.	1038[0n]	University of Natural Resources and Life Sciences
SPDRU199-14	fDbu0102M	*Drusus bureschi*	Bulgaria, Veliko Tarnovo region, Tvardita, Tvardishki Prohod, Ciumerna hut, springs; leg. Ujvarosi, Kolcsar & Torok; 14-Jun-2012	42,786	25,958	658[0n]	541[0n]	362[0n]	837[11n]	346[0n]	1038[0n]	University of Natural Resources and Life Sciences
SPDRU200-14	fDbu0104M	*Drusus bureschi*	Bulgaria, Veliko Tarnovo region, Tvardita, Tvardishki Prohod, Ciumerna hut, springs; leg. Ujvarosi, Kolcsar & Torok; 14-Jun-2012	42,786	25,958	n.a	n.a.	362[0n]	n.a.	n.a.	1038[0n]	University of Natural Resources and Life Sciences
SPDRU201-14	fDbu0105M	*Drusus bureschi*	Bulgaria, Veliko Tarnovo region, Tvardita, Tvardishki Prohod, Ciumerna hut, springs; leg. Ujvarosi, Kolcsar & Torok; 14-Jun-2012	42,786	25,958	658[0n]	541[0n]	362[0n]	n.a.	n.a.	1038[0n]	University of Natural Resources and Life Sciences
SPDRU202-14	fDbu0106m	*Drusus bureschi*	Bulgaria, Veliko Tarnovo region, Tvardita, Tvardishki Prohod, Ciumerna hut, springs; leg. Ujvarosi, Kolcsar & Torok; 14-Jun-2012	42,786	25,958	658[0n]	541[0n]	362[0n]	845[3n]	346[0n]	1037[0n]	University of Natural Resources and Life Sciences
SPDRU203-14	fDbu0109M	*Drusus bureschi*	Bulgaria, Veliko Tarnovo region, Tvardita, Tvardishki Prohod, Ciumerna hut, springs; leg. Ujvarosi, Kolcsar & Torok; 14-Jun-2012	42,786	25,958	n.a.	n.a.	362[0n]	n.a.	n.a.	1038[0n]	University of Natural Resources and Life Sciences
SPDRU231-14	fDdd0801M	*Drusus discolor*	Montenegro, Brodavac, right tributary of Perucica; leg. A. Previsic; 10-Jul-2013	42,6859	19,7364	658[0n]	541[0n]	362[0n]	848[0n]	346[0n]	1038[0n]	University of Natural Resources and Life Sciences
SPDRU232-14	fDdd0802F	*Drusus discolor*	Montenegro, Brodavac, right tributary of Perucica; leg. A. Previsic; 10-Jul-2013	42,6859	19,7364	658[0n]	541[0n]	362[0n]	848[0n]	346[0n]	1038[0n]	University of Natural Resources and Life Sciences
SPDRU235-14	fDdi0101M	*Drusus discophoroides*	Greece, Central Macedonia, Serres, Vyroneia, Beabies; leg. Gordon Ramel; 02-May-2008	41,3167	23,2333	658[0n]	541[0n]	362[0n]	840[8n]	346[0n]	1038[0n]	University of Natural Resources and Life Sciences
SPDRU236-14	fDdi0102M	*Drusus discophoroides*	Greece, Central Macedonia, Serres, Vyroneia, Beabies; leg. Gordon Ramel; 02-May-2008	41,3167	23,2333	658[0n]	490[0n]	362[0n]	836[12n]	346[0n]	999[1n]	University of Natural Resources and Life Sciences
SPDRU243-14	fDds0110M	*Drusus discophorus*	Macedonia, Jablanica Mt, spring of the stream near Labunisko Lake; leg. Kucinic, Krpac, Cukusic; 29-May-2013			658[0n]	474[0n]	360[0n]	848[0n]	346[0n]	1038[0n]	University of Natural Resources and Life Sciences
SPDRU244-14	fDds0111M	*Drusus discophorus*	Macedonia, Jablanica Mt, spring of the stream near Labunisko Lake; leg. Kucinic, Krpac, Cukusic; 29-May-2013			658[0n]	n.a.	360[0n]	848[0n]	346[0n]	1038[0n]	University of Natural Resources and Life Sciences
SPDRU245-14	fDds0112F	*Drusus discophorus*	Macedonia, Jablanica Mt, spring of the stream near Labunisko Lake; leg. Kucinic, Krpac, Cukusic; 29-May-2013			658[0n]	541[0n]	360[0n]	848[0n]	346[0n]	1038[0n]	University of Natural Resources and Life Sciences
SPDRU315-14	fDmy0101M	*Drusus muranyorum*	Greece, Rhodopes, Nea Santa; leg. Balint; 10-Apr-2012	41,128	25,887	658[0n]	541[0n]	362[0n]	848[0n]	346[0n]	1038[0n]	University of Natural Resources and Life Sciences
SPDRU316-14	fDmy0103M	*Drusus muranyorum*	Greece, Rhodopes, Nea Santa; leg. Balint; 20-Apr-2012	41,128	25,887	658[0n]	0[0n]	362[0n]	848[0n]	346[0n]	1038[0n]	University of Natural Resources and Life Sciences
SPDRU317-14	fDmy0104M	*Drusus muranyorum*	Greece, Rhodopes, Nea Santa; leg. Balint; 20-Apr-2012	41,128	25,887	658[0n]	541[0n]	362[0n]	848[0n]	346[0n]	1038[0n]	University of Natural Resources and Life Sciences
SPDRU318-14	fDmy0105M	*Drusus muranyorum*	Greece, Rhodopes, Nea Santa; leg. Balint; 20-Apr-2012	41,128	25,887	n.a.	n.a.	362[0n]	n.a.	n.a.	1038[0n]	University of Natural Resources and Life Sciences
SPDRU324-14	fDos0102M	*Drusus osogovicus*	Bulgaria, Kjustendil region: Osogovo Mts., Bogoslov, Osogovska hut surroundings; leg. Ujvarosi, Kolcsar & Torok; 11-Jun-2012	42,1967	22,6231	658[0n]	541[0n]	362[0n]	810[0n]	346[0n]	1037[1n]	University of Natural Resources and Life Sciences
SPDRU326-14	fDos0105M	*Drusus osogovicus*	Bulgaria, Kjustendil region: Osogovo Mts., Bogoslov, Osogovska hut surroundings; leg. Ujvarosi, Kolcsar & Torok; 11-Jun-2012	42,1967	22,6231	658[0n]	541[0n]	362[0n]	848[0n]	346[0n]	1036[2n]	University of Natural Resources and Life Sciences
SPDRU327-14	fDos0106M	*Drusus osogovicus*	Bulgaria, Kjustendil region: Osogovo Mts., Bogoslov, Osogovska hut surroundings; leg. Ujvarosi, Kolcsar & Torok; 11-Jun-2012	42,1967	22,6231	658[0n]	541[0n]	362[0n]	847[1n]	346[0n]	1035[3n]	University of Natural Resources and Life Sciences
SPDRU328-14	fDos0108M	*Drusus osogovicus*	Bulgaria, Kjustendil region: Osogovo Mts., Bogoslov, Osogovska hut surroundings; leg. Ujvarosi, Kolcsar & Torok; 11-Jun-2012	42,1967	22,6231	658[0n]	541[0n]	362[0n]	848[0n]	346[0n]	1038[0n]	University of Natural Resources and Life Sciences
SPDRU329-14	fDos0118F	*Drusus osogovicus*	Bulgaria, Kjustendil region: Osogovo Mts., Bogoslov, Osogovska hut surroundings; leg. Ujvarosi, Kolcsar & Torok; 11-Jun-2012	42,1967	22,6231	658[0n]	541[0n]	362[0n]	848[0n]	346[0n]	1038[0n]	University of Natural Resources and Life Sciences
SPDRU339-14	fDpo0101M	*Drusus popovi*	Bulgaria, Montana, Western Central Balkan, Gintsi, Petrohanski Prohod, spring; leg. Ujvarosi, Kolcsar & Torok; 11-Jun-2012	43,114	23,1123	658[0n]	n.a.	362[0n]	n.a.	n.a.	1038[0n]	University of Natural Resources and Life Sciences
SPDRU340-14	fDpo0102M	*Drusus popovi*	Bulgaria, Montana, Western Central Balkan, Gintsi, Petrohanski Prohod, spring; leg. Ujvarosi, Kolcsar & Torok; 11-Jun-2012	43,114	23,1123	658[0n]	541[0n]	362[0n]	848[0n]	n.a.	1037[1n]	University of Natural Resources and Life Sciences
SPDRU342-14	fDpo0201F	*Drusus zivici*	Serbia, Stara Planina Mts, spring of the Tovarnicka river; leg. Kucinic, Bjelanovic, Zivic; 19-Jun-2013			658[0n]	541[0n]	362[0n]	848[0n]	346[0n]	1037[1n]	University of Natural Resources and Life Sciences
SPDRU343-14	fDpo0202M	*Drusus zivici*	Serbia, Stara Planina Mts, spring of the Tovarnicka river; leg. Kucinic, Bjelanovic, Zivic; 19-Jun-2013			658[0n]	541[0n]	362[0n]	n.a.	346[0n]	1018[20n]	University of Natural Resources and Life Sciences
SPDRU344-14	fDpo0203M	*Drusus zivici*	Serbia, Stara Planina Mts, spring of the Tovarnicka river; leg. Kucinic, Bjelanovic, Zivic; 19-Jun-2013			658[0n]	541[0n]	362[0n]	846[2n]	346[0n]	1038[0n]	University of Natural Resources and Life Sciences
SPDRU345-14	fDpo0204L	*Drusus zivici*	Serbia, Stara Planina Mts, spring of the Tovarnicka river; leg. Kucinic, Bjelanovic, Zivic; 19-Jun-2013			658[0n]	541[0n]	362[0n]	783[5n]	346[0n]	1038[0n]	University of Natural Resources and Life Sciences
SPDRU346-14	fDpo0205L	*Drusus zivici*	Serbia, Stara Planina Mts, spring of the Tovarnicka river; leg. Kucinic, Bjelanovic, Zivic; 19-Jun-2013			658[0n]	541[0n]	362[0n]	845[3n]	346[0n]	1038[0n]	University of Natural Resources and Life Sciences
SPDRU347-14	fDpo0301M	*Drusus zivici*	Serbia, Stara Planina Mts, spring of the Rekicka river; leg. Kucinic, Bjelanovic, Zivic; 19-Jun-2013			658[0n]	541[0n]	362[0n]	846[2n]	346[0n]	1009[0n]	University of Natural Resources and Life Sciences
SPDRU348-14	fDpo0302M	*Drusus zivici*	Serbia, Stara Planina Mts, spring of the Rekicka river; leg. Kucinic, Bjelanovic, Zivic; 19-Jun-2013			658[0n]	541[0n]	362[0n]	n.a.	346[0n]	1038[0n]	University of Natural Resources and Life Sciences
SPDRU349-14	fDpo0401F	*Drusus zivici*	Serbia, Stara Planina Mts, spring Kluderske vode; leg. Kucinic, Bjelanovic, Zivic; 20-Jun-2013			658[0n]	541[0n]	325[0n]	848[0n]	346[0n]	1036[2n]	University of Natural Resources and Life Sciences
SPDRU350-14	fDpo0501F	*Drusus zivici*	Serbia, Stara Planina Mts, spring of the Javorska river; leg. Kucinic, Bjelanovic, Zivic; 20-Jun-2013			658[0n]	541[0n]	362[0n]	631[1n]	346[0n]	1038[0n]	University of Natural Resources and Life Sciences
SPDRU541-15	fDsp4301M	*Drusus dardanicus*	Kosovo, Llap catchment; leg. Ibrahimi; 11-May-2014			658[0n]	541[0n]	362[0n]	850[0n]	345[0n]	n.a.	University of Natural Resources and Life Sciences
SPDRU542-15	fDsp4302M	*Drusus dardanicus*	Kosovo, Llap catchment; leg. Ibrahimi; 11-May-2014			658[0n]	541[0n]	362[0n]	850[0n]	345[0n]	1000[0n]	University of Natural Resources and Life Sciences
SPDRU031-14	fEda0201M	*Ecclisopteryx keroveci*	Montenegro, Brodavac, right tributary of Perucica; leg. Milisa;	42,6859	19,7364	658[0n]	541[0n]	362[0n]	842[6n]	346[0n]	1038[0n]	University of Natural Resources and Life Sciences
SPDRU032-14	fEda0202F	*Ecclisopteryx keroveci*	Montenegro, Brodavac, right tributary of Perucica; leg. Milisa;	42,6859	19,7364	658[0n]	541[0n]	362[0n]	846[2n]	346[0n]	1038[0n]	University of Natural Resources and Life Sciences
SPDRU003-14	fEda0801M	*Ecclisopteryx dalecarlica*	Norway, Hedmark, E 9.96412, N, Hedmark (HEN), Folldal, Streitlie; leg. Andersen;	62,095	9,964	658[0n]	541[0n]	362[0n]	841[7n]	346[0n]	1038[0n]	University of Natural Resources and Life Sciences
SPDRU004-14	fEda0802F	*Ecclisopteryx dalecarlica*	Norway, Hedmark, E 9.96412, N, Hedmark (HEN), Folldal, Streitlie; leg. Andersen;	62,095	9,964	658[0n]	541[0n]	362[0n]	842[6n]	346[0n]	1038[0n]	University of Natural Resources and Life Sciences
SPDRU496-14	fMelaus0101M	*Melampophylax austriacus*	Austria, spring Schwarze Sulm; leg. Graf; 20-Oct-2013	46,8106	14,9931	658[0n]	541[0n]	361[0n]	842[6n]	n.a.	1038[0n]	University of Natural Resources and Life Sciences
SPDRU497-14	fMelaus0102F	*Melampophylax austriacus*	Austria, spring Schwarze Sulm; leg. Graf; 20-Oct-2013	46,8106	14,9931	658[0n]	n.a.	361[0n]	843[5n]	n.a.	1038[0n]	University of Natural Resources and Life Sciences
